# Mutations in human AID differentially affect its ability to deaminate cytidine and 5-methylcytidine in ssDNA substrates *in vitro*

**DOI:** 10.1038/s41598-017-03936-x

**Published:** 2017-06-20

**Authors:** Lucyna Budzko, Paulina Jackowiak, Karol Kamel, Joanna Sarzynska, Janusz M. Bujnicki, Marek Figlerowicz

**Affiliations:** 1 Institute of Bioorganic Chemistry, Polish Academy of Sciences, Noskowskiego 12/14, 61-704 Poznan, Poland; 2 Laboratory of Bioinformatics and Protein Engineering, International Institute of Molecular and Cell Biology in Warsaw, Trojdena 4, 02-109 Warsaw, Poland; 3 Laboratory of Bioinformatics, Institute of Molecular Biology and Biotechnology, Faculty of Biology, Adam Mickiewicz University, Umultowska 89, 61-614 Poznan, Poland; 4 Institute of Computing Science, Poznan University of Technology, Piotrowo 3A, 60-965 Poznan, Poland

## Abstract

Activation-induced cytidine deaminase (AID) is known for its established role in antibody production. AID induces the diversification of antibodies by deaminating deoxycytidine (C) within immunoglobulin genes. The capacity of AID to deaminate 5-methyldeoxycytidine (5 mC) and/or 5-hydroxymethyldeoxycytidine (5 hmC), and consequently AID involvement in active DNA demethylation, is not fully resolved. For instance, structural determinants of AID activity on different substrates remain to be identified. To better understand the latter issue, we tested how mutations in human AID (hAID) influence its ability to deaminate C, 5 mC, and 5 hmC *in vitro*. We showed that each of the selected mutations differentially affects hAID’s ability to deaminate C and 5 mC. At the same time, we did not observe hAID activity on 5 hmC. Surprisingly, we found that the N51A hAID mutant, with no detectable activity on C, efficiently deaminated 5 mC, which may suggest different requirements for C and 5 mC deamination. Homology modeling and molecular dynamics simulations revealed that the pattern of enzyme-substrate recognition is one of the important factors determining enzyme activity on C and 5 mC. Consequently, we have proposed mechanisms that explain why wild type hAID more efficiently deaminates C than 5 mC *in vitro* and why 5 hmC is not deaminated.

## Introduction

Activation-induced cytidine deaminase (AID) is a member of a zinc-dependent AID/APOBEC deaminase family. This family includes enzymes that deaminate deoxycytidine/cytidine to deoxyuridine/uridine in DNA and/or RNA chains^1^. AID is expressed in activated germinal center B cells, where it participates in secondary antibody diversification^2, 3^. AID initiates both somatic hypermutation (SHM) and class switch recombination (CSR) by the deamination of deoxycytidine (C) to deoxyuridine (U) within, respectively, the variable and switch regions of actively transcribed immunoglobulin genes. As a result, a repertoire of antibodies highly specific to an antigen and with different effector functions is produced^4, 5^.

AID acts exclusively on ssDNA (generated during transcription) and deaminates C preferentially within WRCY or WRC motifs (W = A/T; R = A/G; Y = C/T)^6–9^. Although the mechanisms of DNA scanning and C residue recognition by AID have been a matter of intensive studies both issues have still remained not fully resolved^8–13^. The data recently published by Senavirathne *et al*. revealed substantial differences between the mechanism of co-transcriptional, AID-mediated deamination of locally unwound dsDNA (when AID is constrained to act within a small, 8 to 12 nt transcription bubble) and the mechanism of AID-mediated deamination of ssDNA (when AID is unconstrained and can act on the whole molecule)^14^. Senavirathne and co-workers have shown that in the first case, AID binds to the transcribed DNA (to the transiently exposed single-stranded region in a transcription bubble) and translocates unidirectionally in concert with RNA polymerase. In the second case, AID binds ssDNA essentially randomly and scans it in random bidirectional short sliding or hopping movements. Moreover, Senavirathne and co-workers have observed that AID scans multiple times along the entire length of the ssDNA during a single binding event. The mean lifetime for AID bound to ssDNA is ∼5 min^14^.

Although both SHM and CSR depend on AID deamination activity, the amino acid mutations that alter the latter may differentially affect these processes (Supplementary Table S1). This phenomenon can be readily explained if the mutations are located within C- and/or N-terminal regions of AID because it has been postulated that the termini of this enzyme bind protein partners that recruit AID to specific genomic regions to trigger SHM (N terminus) or CSR (C terminus)^15, 16^. The problem is more complicated if one considers mutations located close to the AID catalytic center. For example, AID carrying a single N51 to A mutation (N51A mutant) has been shown to have no detectable activity *in vitro* ^17, 18^. Accordingly, one of the studies revealed that SHM was abolished in cells harboring this mutation; unexpectedly, however, CSR was still observed^17^.

In addition, the proposed capacity of AID to deaminate 5-methyldeoxycytidine (5 mC) to deoxythymidine (T) has suggested that this enzyme might be involved in active DNA demethylation. Genome methylation is one of the epigenetic mechanisms that have a profound effect on gene expression and genome stability^19^. DNA methylation occurs primarily at C and leads to the formation of 5 mC. Although enzymes that catalyze DNA methylation in mammals are well characterized (DNMT proteins)^20^, the existence of enzymes involved in methyl group removal remains an enigma. The genome is passively demethylated during replication when non-methylated Cs are incorporated into newly synthesized DNA. Additionally, several mechanisms of active genome demethylation have been proposed^21, 22^. According to these mechanisms 5 mC undergoes enzymatic conversion to another modified nucleotide that is subsequently processed by DNA repair machinery. The latter replaces this modified nucleotide with C, and thus, the epigenetic mark is removed^23–25^. There are three possible scenarios of 5 mC modification and exchange into C^21, 26^: (i) direct deamination of 5 mC to T by AID/APOBEC deaminase(s), followed by processing of the T-G mismatch by base excision repair (BER) machinery^27, 28^; (ii) oxidation of 5 mC to 5 hmC catalyzed by TET proteins, followed by deamination of 5 hmC to 5-hydroxymethyldeoxyuracil (5 hmU) by AID/APOBEC deaminase(s) and 5 hmU removal by BER^28, 29^; and (iii) oxidation of 5 mC to 5 hmC catalyzed by TET proteins, followed by iterative oxidation that leads to the formation of 5-formyldeoxycytidine (5 fC) and then 5-carboxyldeoxycytidine (5caC). The latter analogs of C can also be processed by BER^30^.

It has been proposed that AID is involved in active DNA demethylation in several *in vivo* systems (reviewed in ref. 31), such as *Danio rerio* ^32^ and *Xenopus leavis* embryos^33^, the heterokaryon system^34^, mouse primordial germ cells^35^ and mouse stem cells^36^, mainly upon AID overexpression or its ectopic expression. It has also been shown that AID may promote 5 hmC demethylation in both cultured human cells and the adult mouse brain^29^. AID has been proven to deaminate 5 mC *in vitro* ^6, 27, 37, 38^. In contrast, the deamination activity on 5 hmC *in vitro* has not been confirmed^37, 39^.

Although a substantial number of reports support the involvement of AID in active DNA demethylation, this idea is still highly controversial^31, 40^. For example, phenotypes of humans with non-functional AID have not been linked, so far, to any epigenetic process and the only observed impairments are in the immune system^40, 41^. Therefore, one can hypothesize that AID, if at all responsible for active genome demethylation in mammals, is not the only factor implicated in this process and that AID may have a more subtle role, e.g., it may participate in the demethylation of only certain loci. However, data showing that AID-dependent demethylation actually occurs under physiological conditions are lacking. Moreover, several *in vitro* studies have shown that AID deaminates 5 mC less efficiently than C^6, 37, 38, 42, 43^ and that 5 hmC is not deaminated by this enzyme^37, 39^. The analysis of AID activity on a series of modified substrates has suggested that one of the causes of the lower activity of AID on 5 mC (relative to non-methylated substrate) and the lack of the activity on 5 hmC is a size restriction against the 5-substituted C imposed by the enzyme’s catalytic pocket^37, 39^. Consequently, it has been postulated that, due to a steric hindrance, AID may only occasionally deaminate 5 mC^42, 43^.

To better understand the structural determinants of hAID activity on different substrates, we tested how selected mutations affect hAID ability to deaminate C and its 5-substituted derivatives *in vitro*. We found that all examined hAID variants were able to deaminate 5 mC, and all of them failed to deaminate 5 hmC. Notably, we observed a lack of correlation between the individual variants’ ability to deaminate C and 5 mC. The lack of correlation was particularly evident for the N51A mutant found to be active on 5 mC and have no detectable activity on C. The observed decoupling of hAID activity on methylated and non-methylated substrates revealed that the structural determinants of C and 5 mC deamination by hAID can be different. To identify these determinants, we used computational methods to generate models of complexes that hAID and its N51A mutant form with C and 5 mC. Comparative analysis of the models suggested that the size restriction against the 5-substituted C was not responsible for the observed effects. The data presented herein indicated, for the first time, significant differences between the molecular mechanisms of C and 5 mC recognition by hAID.

## Results

### hAID mutants exhibit different and non-correlated deaminase activities on non-methylated and methylated substrates and have no detectable activity on hydroxymethylated substrate

To identify the structural determinants of hAID activity on different substrates, we decided to obtain recombinant wild type enzyme (wt hAID) as well as its mutants, and to test their ability to deaminate C and 5-substituted C *in vitro*. Of a number of well-characterized mutants, we selected three: R50A, N51A and R190X (Table 1). In R50A and N51A, single amino acid alterations were located in the vicinity of the catalytic center. R190X had a 9 amino acid truncation at the very end of the C-terminal region. It was previously reported that, *in vitro*, the R50A mutant exhibited decreased deaminase activity on C^17^, whereas N51A failed to show this activity^17, 18^, and R190X was more active than wt hAID^15^. Moreover, the *in vitro* activities of R50A, N51A and R190X did not correlate with the efficiency of either SHM or CSR observed in cells harboring corresponding mutations (see Table 1). For example, the efficiency of CSR in R50A- and N51A-cells was 130% and 55%, respectively, and in R190X-cells CSR was nearly absent (7%). Importantly, the deaminase activities of the selected mutants on substrates containing 5-substituted C have not yet been determined.

**Table 1:** The effect of selected mutations on AID’s ability to induce SHM and CSR *in vivo* and to deaminate C *in vitro*.

AID mutants	SHM or CSR efficiency *in vivo* (% in relation to wt AID-cells)*		deamination activity on C *in vitro* (% in relation to wt AID)*	source
	CSR	SHM		
R50A	130%	25%	20%	17
N51A	55%	0%	0%	
R190X	7%	75%	≫100%	15, 76

^*^Approximate values are given.

The wt hAID enzyme and selected mutants were obtained in a bacterial expression system (see Methods). To determine the amount of active hAID in our preparations, we used a previously published active site titration method^13, 44^. This method allowed us to calculate the percentage of active hAID to be 10–14% (Supplementary Fig. S1). We also performed a detailed characterization of both: (i) recombinant hAID protein (MALDI-TOF and western blot analyses, a test of inhibition of hAID’s activity by 1,10-phenanthroline, a test of inhibition of hAID activity by tetrahydrouridine) and (ii) deamination products after each step of hAID activity assay (see Methods and Supplementary Methods). The deaminase activities of the recombinant hAID variants were tested in reactions involving 5′-radiolabeled 80-nucleotide ssDNA that in the middle contained only one C or its analog (5 hmC or 5 mC) within the AGCT, AG5 hmCT or AG5 mCT motifs. In these experiments we used three previously published assays^37, 38, 45–47^: treatment with uracil-DNA glycosylase (UDG), human single-strand-selective monofunctional uracil-DNA glycosylase (hSMUG) or thermostable thymine DNA glycosylase (TDG) followed by alkaline hydrolysis of the abasic site (see Methods). In control reactions, we used oligonucleotides that instead of C, 5 hmC or 5 mC, contained the products of their deamination: U, 5 hmU or T, respectively. The deamination of C or its analog resulted in the formation of a 40-nucleotide-long ^32^P-labeled product. In each set of experiments, the deamination activities of mutants were determined in relation to wt hAID.

**Figure 1:**
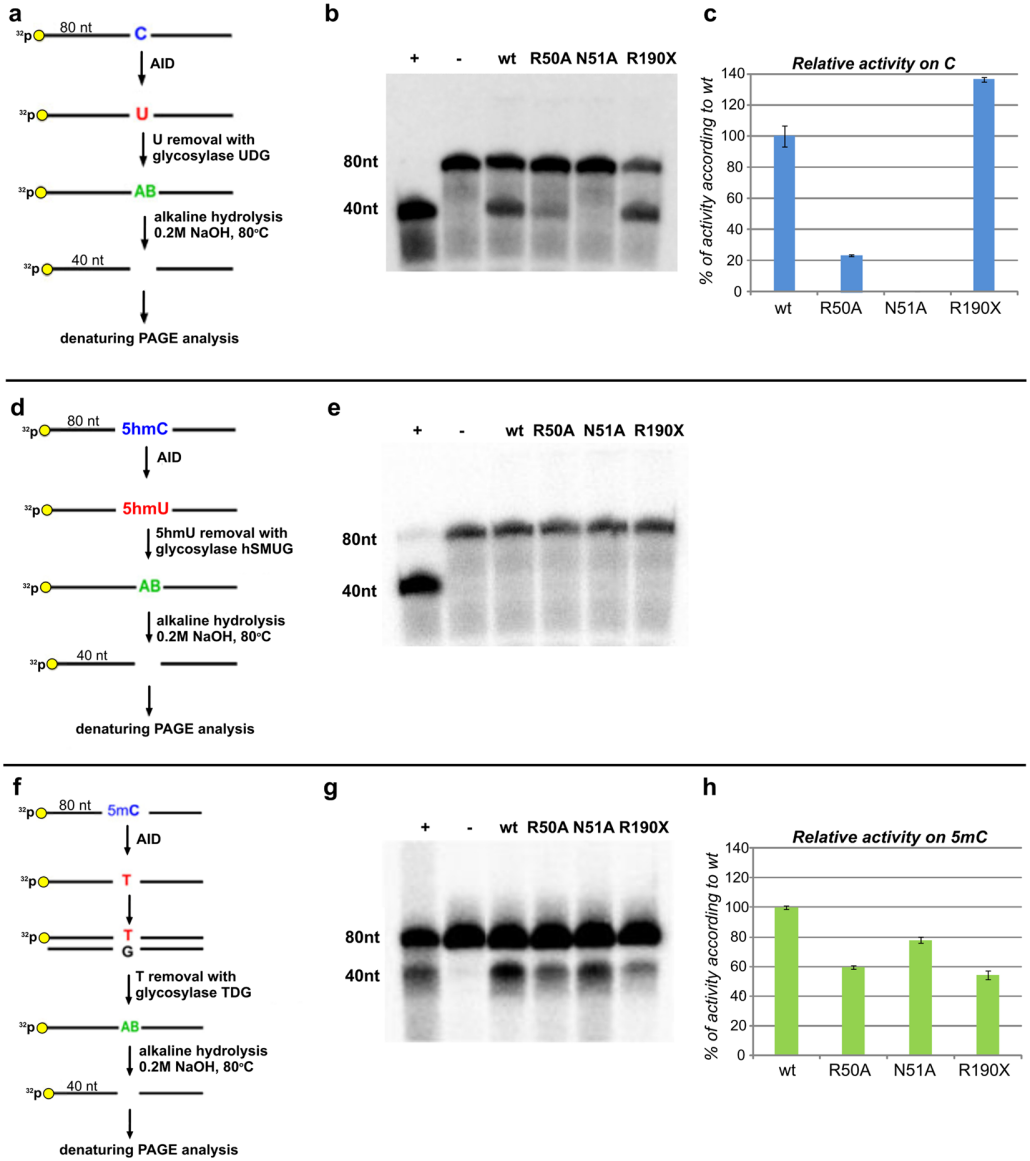
The deaminase activity of the tested hAID variants (wt, R50A, N51A, R190X) on C (**a**–**c**, upper panel), 5 hmC (**d**,**e**, middle panel) and 5 mC (**f**–**h**, bottom panel). (**a**,**d**,**f**) Schematic depiction of hAID activity assays applied for the analyses of C, 5 hmC and 5 mC deamination, respectively (see Methods). (**b**,**e**,**g**) Denaturing PAGE analysis of the products of deamination of C, 5 hmC and 5 mC, respectively. In the case of deamination, a 40-nucleotide-long product was expected. Positive and negative control reactions are indicated by “+” or “−”, respectively. The cropped gel images are displayed. Full-length gel images are shown in Supplementary Fig. S6. (**c**,**h**) Relative deaminase activities of hAID mutants on C or 5 mC. The activities are expressed as a percentage relative to the activity of wt hAID (considered as 100%).

**Figure 2:**
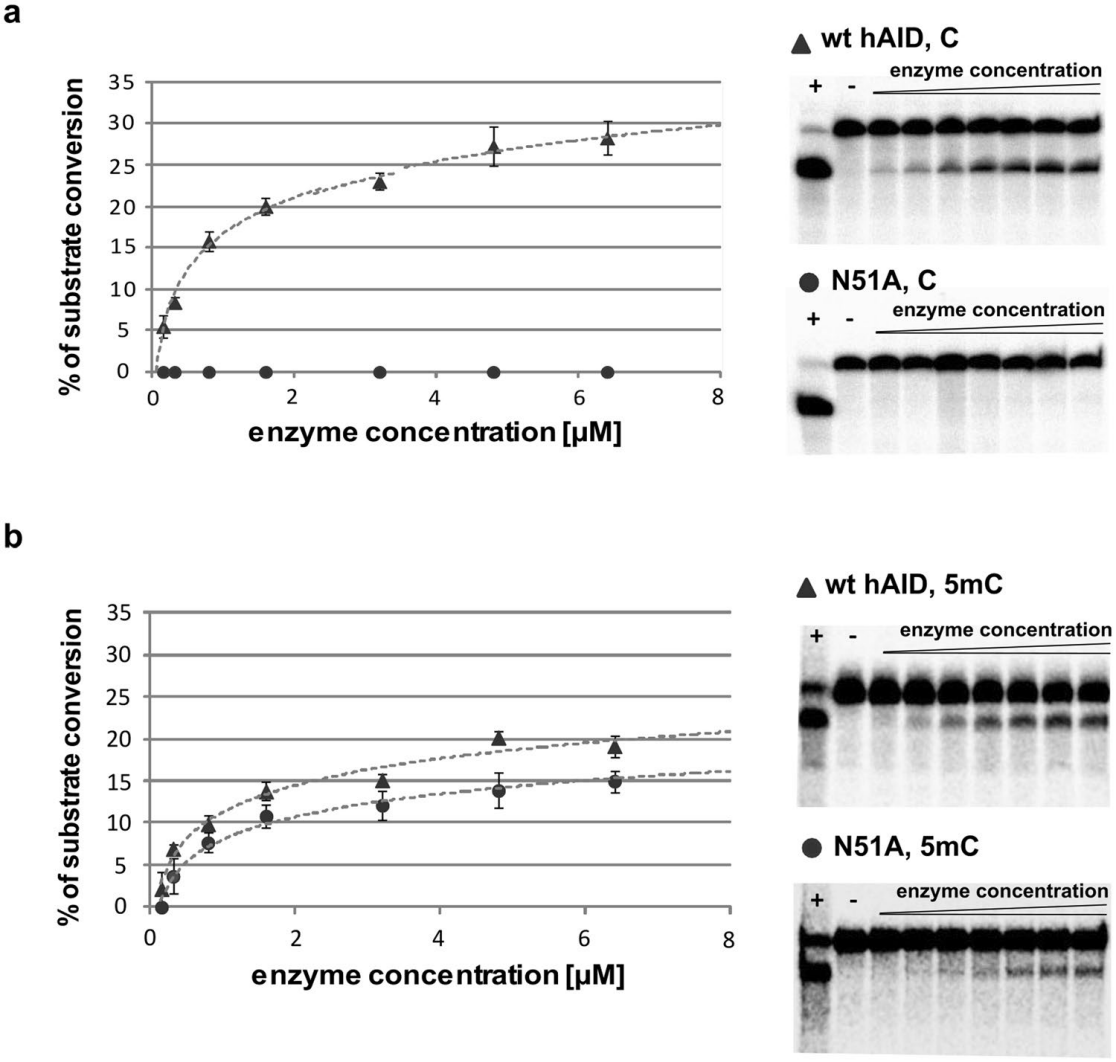
Analyses of enzyme’s concentration-dependent deamination of C (**a**) and 5 mC (**b**) containing substrates by wt hAID and N51A mutant. The graph presents the dependence between the yield of C/5 mC deamination reactions and enzyme concentration (wt hAID (triangles) and N51A mutant (circles)). UDG-coupled and TDG-coupled deamination assays (see Methods) were used for C and 5 mC substrates, respectively. Error bars represent standard deviation from the mean of at least three independent replicates. Representative PAGE analyses of the products of deamination reaction are shown on the right. Positive and negative control reactions are indicated by “+” or “−”, respectively. The cropped gel images are displayed. Full-length gel images are shown in Supplementary Fig. S7.

In the first set of experiments, we determined the activity of wt hAID and its mutants on the 80-nucleotide ssDNA containing the AGCT motif. We observed that hAID exhibited a robust deaminase activity, the truncated mutant R190X was hyperactive and showed nearly 140% activity, R50A displayed approximately 20% activity, and N51A had no detectable activity (Fig. 1a–c, upper panel). Notably, these results agreed with characteristics of these mutants obtained in other expression systems (Table 1). In the next sets of experiments, we used the same hAID variants and analogous oligonucleotides of the same length, which instead of C contained 5 mC or 5 hmC. As shown in Fig. 1(1d,e, middle panel), neither wt hAID nor its mutants R50A, N51A, R190X had detectable activities on 5 hmC, and all of them deaminated 5 mC (Fig. 1f–h, bottom panel). Surprisingly, the deaminase activities of the tested hAID variants on C did not correlate with their deaminase activities on 5 mC (Supplementary Fig. S2). The R190X mutant, showing ~140% activity on C, exhibited only ~55% of the wt deaminase activity on 5 mC. For the R50A mutant, we observed a considerable decrease in deaminase activity; however, this decrease was less pronounced for 5 mC (to 60%) than for the non-modified substrate (to nearly 20%). Unexpectedly, the N51A mutant, with no detectable activity on C, exhibited robust, activity on 5 mC (~78% of the wt hAID activity). To further support this observation we analyzed how the concentration of the enzyme influences the yield of the C and 5 mC deamination product generated by wt hAID and N51A mutant. In the case of C the activity of N51A mutant was below detection level across the enzyme’s concentration range. In the case of 5mC the same differences between the activity of wt hAID and N51A mutant were observed for all tested concentrations of the enzymes (Fig. 2).

**Figure 3:**
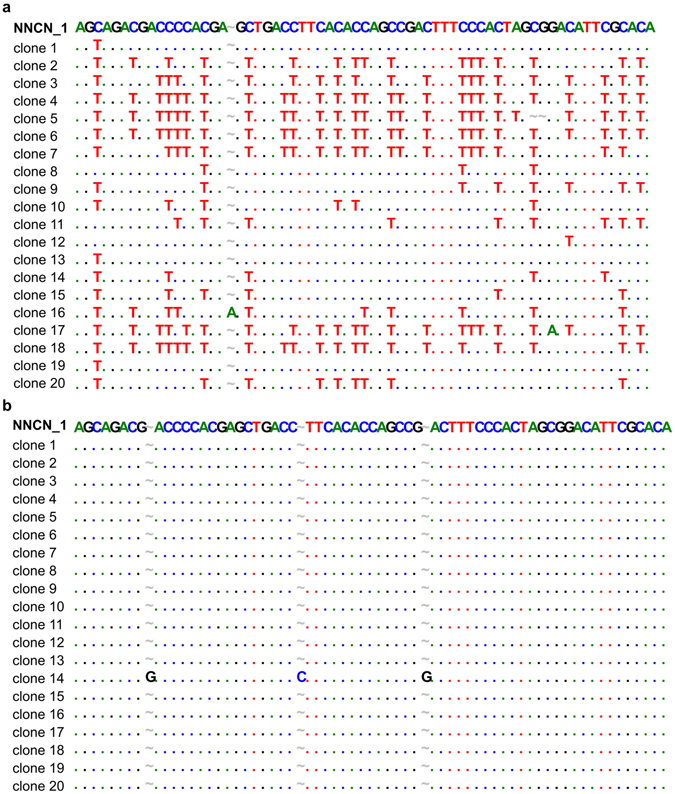
Representative results obtained during the analysis of wt hAID (**a**) or N51A mutant (**b**) deaminase activities on all possible combinations of NNCN sequences (20 clone sequences for NNCN_1 oligonucleotide; results for NNCN_2, 3, and 4 oligonucleotides are shown in Supplementary Fig. S3). The first line of each alignment contains the sequence of the NNCN_1 oligonucleotide untreated with hAID. Substitutions are indicated by one-letter symbols corresponding to a particular nucleotide. Positions identical to those in the untreated sequence are marked by dots. Deletions/insertions presumably caused by the used polymerase are marked by “~”. Flanking sequences – 20-nt-long PCR primer binding sites are not shown in the figure.

The lack of deaminase activity of N51A on C could indicate that this mutant lost its ability to deaminate C or changed its hot-spot preference. To resolve this issue, we examined the deaminase activity of wt hAID and N51A on all possible NNCN motifs (where N is A, G, C or T). We designed four 104 nucleotide-long ssDNA, each containing 16 out of 64 possible combinations of NNCN motifs flanked by 20 nt-long PCR primer binding sites. Each oligonucleotide was incubated with wt hAID or the N51A mutant and subsequently amplified by PCR with a high-fidelity polymerase that tolerates uracil residues in the template. The PCR products were cloned and sequenced (20 clones for each oligomer). As a result, we found that wt hAID could deaminate C located within different nucleotide contexts (Fig. 3 and Supplementary Fig. S3). The frequency of AID-mediated ssDNA deamination within different NNCN motifs is shown in Supplementary Fig. S3g,h. In contrast, the N51A mutant had no detectable activity on any of the NNCN motifs. Altogether, our data demonstrated that a single N51-to-A mutation located in close vicinity to the putative catalytic pocket abolished the ability of hAID to deaminate C while maintaining the enzyme’s activity on 5 mC.

### The steric effects do not hinder 5 mC deamination by wt hAID and N51A mutant

The lack of correlation between hAID’s ability to deaminate C and 5 mC suggested that there are different structural determinants for non-modified and modified substrates’ recognition and/or binding. This phenomenon was particularly pronounced for the N51A mutant that lost its activity on C and was active on 5 mC. Notably, to date, it has been postulated that the steric hindrance imposed by the catalytic pocket is one of the major factors responsible for the lower efficiency of 5-substituted C deamination compared with C^37, 39^. To test this hypothesis, we examined the interactions of wt hAID and the N51A mutant with non-modified and modified substrates by applying homology modeling and molecular dynamics (MD) simulations. To generate the models of wt hAID and N51A mutant we used structures that were available when we commenced our studies i.e. the structures of the C-terminal catalytic domain of APOBEC3G (PDB entry 3V4K and 3E1U). The generated models were further refined with extensive MD simulations.

**Figure 4:**
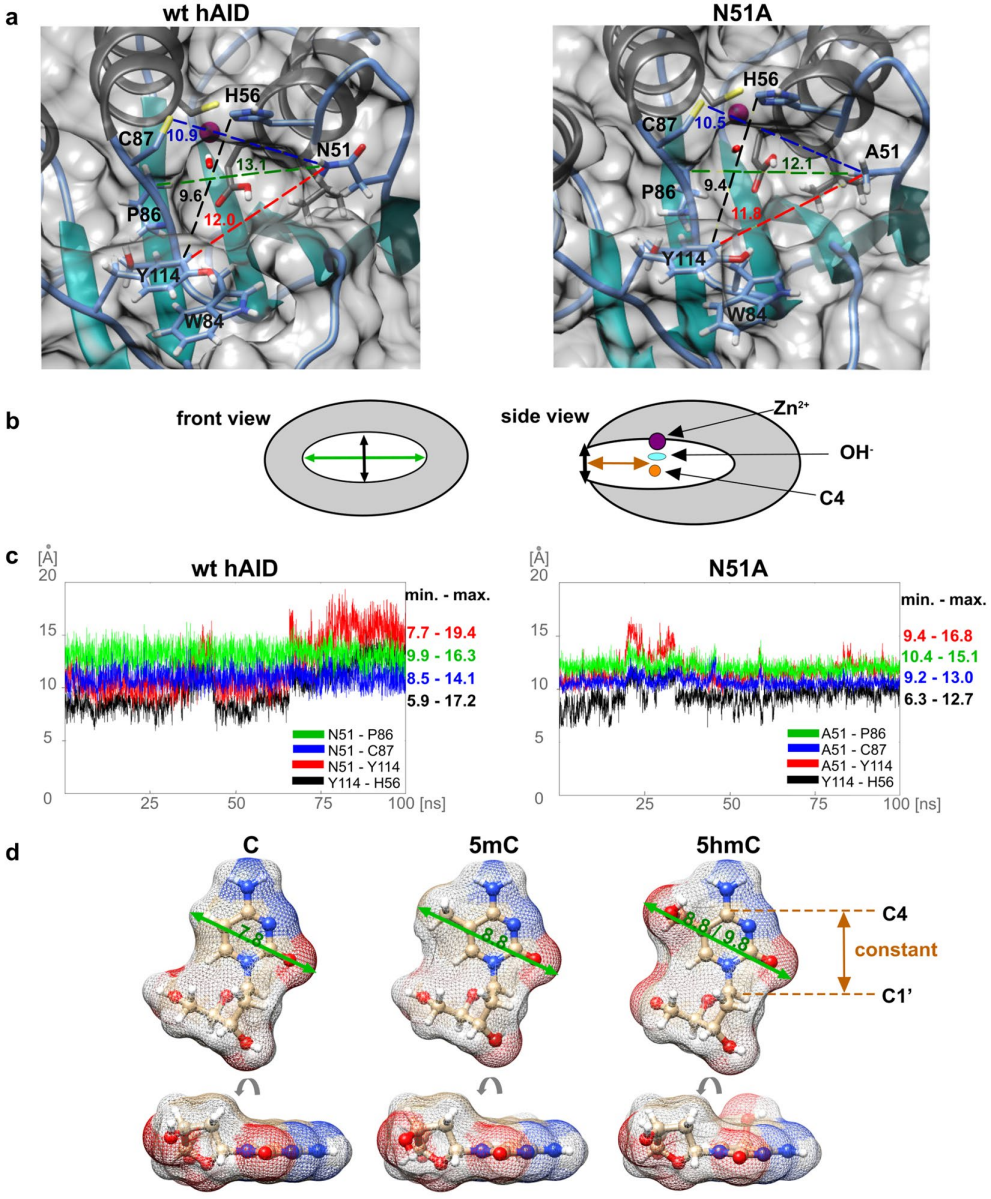
Comparison of the sizes of wt hAID/N51A mutant catalytic pockets with the sizes of modified and non-modified C. (**a**) Catalytic pockets of wt hAID and the N51A mutant. The size of each pocket was defined by the distances between the selected atoms that belong to four pairs of amino acid residues: N51 (A51 in the case of the mutant) and Y114 (red dashed line); N51 (A51) and P86 (green dashed line); N51 (A51) and C87 (blue dashed line); and Y114 and H56 (black dashed line). The value of each distance is indicated and expressed in Å as a mean value from the distances measured for all individual frames of the MD trajectories. The distances were determined between the following heavy atoms: ND2 atom of N51; CB atom of A51; CE2 atom of Y114; CB atom of P86, SG atom of C87, and CE1 atom of H56. (**b**) Front and side views of hAID catalytic pocket. The distance between N51 (A51) and P86, i.e., the distance determining the breadth of a heterocyclic ring that can enter the pocket, is marked by a green arrow. The distance that defines the height of the catalytic pocket (between Y114 and H56) is marked by a black arrow. The depth of the catalytic pocket is marked by an orange arrow. (**c**) Range of changes observed for each distance throughout MD trajectories. The maximal and minimal values of each distance (expressed in Å) are indicated next to the graph. The colors are the same as in panel a. **(d)** The breadths of the heterocyclic rings of modified and non-modified C (green arrows). The value of each breadth is expressed in Å and includes vdW radii. The distance between C1′ and C4 of the heterocyclic ring is constant for each of the substrates (marked by an orange arrow) and corresponds to the depth of the catalytic pockets. A side view of the substrates is also shown. Because of the rotation of the hydroxymethyl group, the ring of 5 hmC is not completely flat.

To assess whether the steric hindrance restricts the hAID ability to deaminate 5-substituted C, we compared the sizes of catalytic pockets of wt hAID and the N51A mutant with the sizes of modified and non-modified cytosines. The size of each pocket was defined by the distances between selected heavy atoms (located at the internal surface of the pocket) that belong to four pairs of amino acid residues: (1) N51 (A51 in the case of the mutant) and Y114; (2) N51 (A51) and P86; (3) N51 (A51) and C87; (4) Y114 and H56 (Fig. 4a). The first three distances took into account the positioning of the N51 residue, whose replacement with A changed the enzymatic properties of hAID. The fourth distance defined the height of the catalytic pocket. Each distance was expressed as the mean value of distances measured for all individual frames of the MD trajectories. In addition, the maximal and minimal values of each distance were determined. As shown in Fig. 4a and c, the distances defining the sizes of the catalytic pockets in wt hAID and N51A mutant were similar to each other and did not change significantly along either MD trajectory. The distance between N51 (A51) and P86, i.e., the distance determining the breadth of a heterocyclic ring that can enter the pocket, was 9.9–16.3 Å in wt hAID and 10.4–15.1 Å in the N51A mutant. Considering the size of C and 5 mC heterocyclic rings, 7.8 Å and 8.8 Å (including van der Waals (vdW) radii), respectively, one can conclude that both should fit even if the pockets of wt hAID and the N51A mutant adopt their minimal sizes (Fig. 4d). Theoretically, 5 hmC should also fit in the catalytic pocket of both wt hAID and the N51A mutant because the width of its heterocyclic ring ranged between 8.8 and 9.8 Å (including vdW radii) due to the rotation of the hydroxyl group. However, there are two additional factors that could preclude 5 hmC deamination. The first is the hydration of the strongly hydrophilic hydroxyl group, which can bind up to five water molecules. A single water molecule adds approximately 2.8 to 3.2 Å^48, 49^ of steric bulk to the size of the hydroxyl group, making the effective size of 5 hmC too large to enter the catalytic pocket. Dehydration, necessary for 5 hmC deamination, would be energetically highly unfavorable. The second factor is the hydrophobic character of the bottom surface of the catalytic pocket. Hypothetically, if dehydrated 5 hmC entered the catalytic pocket, the hydrophobic amino acid residues would force the hydrophilic hydroxyl group to move in the direction of the activated water molecule coordinated by zinc ion^50^ (Supplementary Fig. S4a and b). As a result, a hydrogen bond (H-bond) between the hydroxymethyl group and activated water would form. The postulated mechanism of C deamination presumes a direct nucleophilic attack of the activated water molecule at position 4 of the pyrimidine ring^50^. The formation of the abovementioned H-bond would prevent the nucleophilic attack that initiates the deamination process (Supplementary Fig. S4c and d). Accordingly, we hypothesized that hAID does not deaminate 5 hmC *in vitro* due to the presence of the hydroxyl group, which either is hydrated and 5 hmC cannot enter the catalytic pocket or disturbs the deamination mechanism if 5 hmC enters the pocket.

Our analysis indicated that both wt hAID and the N51A mutant have enough space in their catalytic pockets to bind either methylated or non-methylated cytosine. To further strengthen this conclusion, we docked deoxycytidine bismonophosphate or 5-methyldeoxycytidine bismonophosphate to the obtained models of wt hAID and the N51A mutant and subjected both complexes to 50 ns MD simulations. This procedure confirmed that both substrates fit well in the catalytic pockets and remained inside the pockets throughout the simulations (Supplementary Movies S1–S4). In the case of the non-methylated substrate, we observed an empty space in the proximity of the deoxycytidine C5 atom throughout most of the time of the simulations. In general, our results clearly showed that there was no size restriction against the methyl moiety imposed by catalytic pockets of wt hAID and the N51A mutant. Thus, the steric effects cannot be considered the factors responsible for the different abilities of wt hAID and the N51A mutant to deaminate C and 5 mC.

### Structural determinants of C and 5 mC deamination by wt hAID and N51A mutant

Because the steric effects could not shape the ability of wt hAID or the N51A mutant to deaminate C and 5 mC, we hypothesized that specific changes in the pattern of enzyme-substrate recognition were responsible for the observed effects. If this hypothesis is true, the N51-to-A mutation should eliminate some interactions indispensable for deamination of C and retain/create interactions that ensure the deamination of 5 mC. To identify these key factors affecting the pattern of enzyme-substrate recognition, we performed two series of analyses. The first focused on the interaction networks formed between C/5 mC pyrimidine rings and amino acid residues constituting catalytic pockets of the wt hAID/N51A mutant. The second analysis concentrated on the interaction networks between the wt hAID/N51A mutant and the AGCT/AG5mCT tetranucleotides.

**Figure 5:**
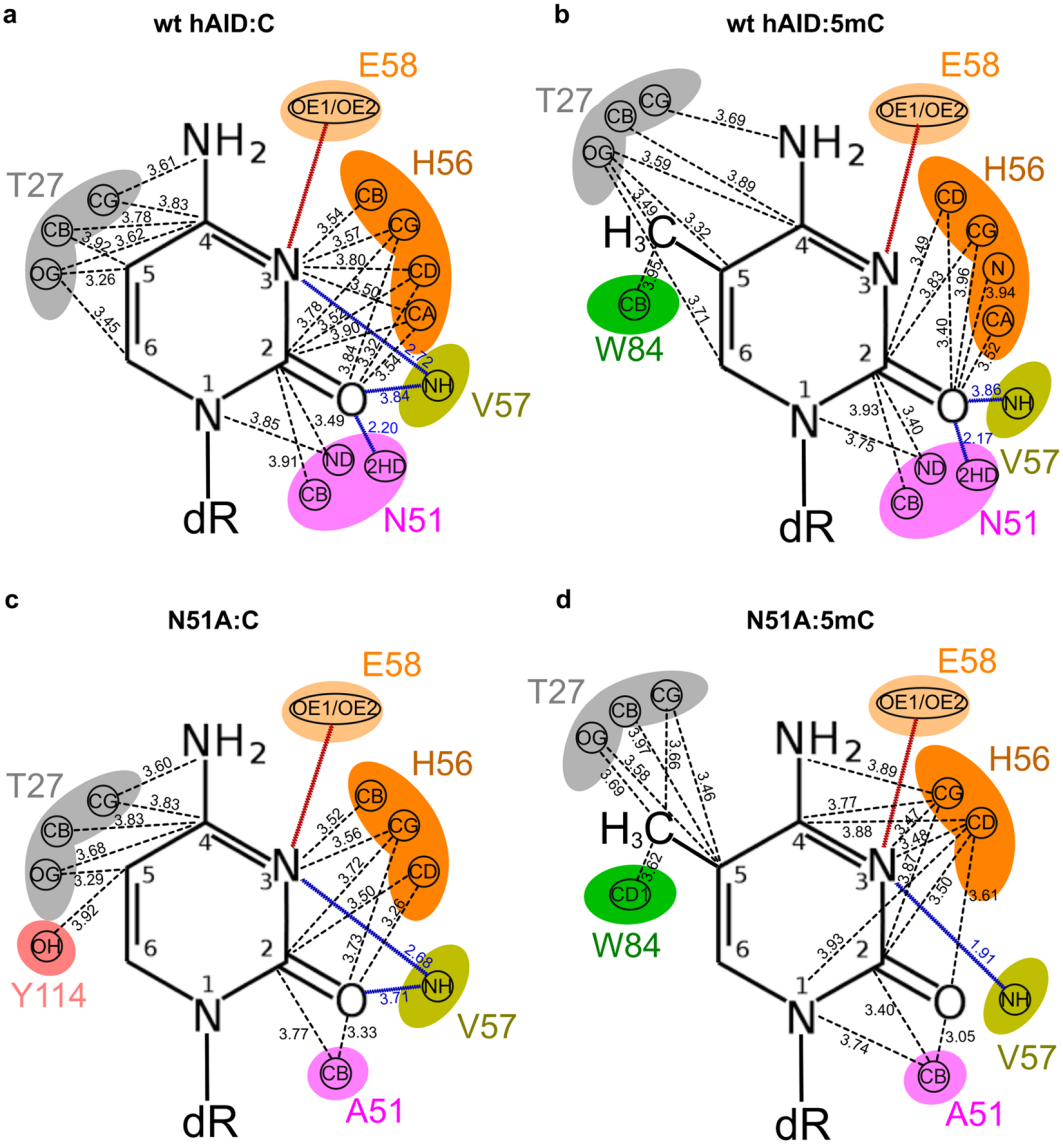
Networks of interactions between C/5 mC pyrimidine rings and wt hAID/N51A mutant catalytic pockets within the four enzyme:substrate complexes: (**a**) wt hAID:C, (**b**) wt hAID:5 mC, (**c**) N51A:C, and (**d**) N51A:5 mC. Different colors represent individual amino acid residues that form catalytic pockets of the tested enzymes. Atoms involved in the particular interactions with the substrate are indicated. VdW interactions are marked by dashed lines. H-bonds are marked by blue lines. The red line represents a hypothetical H-bond whose existence is determined based on the postulated mechanism of the deamination reaction. The distances between interacting atoms are expressed in Å as mean values from the distances measured for all individual frames of the MD trajectories.

The first analysis, involving the four above-described models of complexes formed by wt hAID/N51A mutant and C/5 mC bismonophosphates, showed that the number of intermolecular interactions was the highest (24) in the wt hAID:C complex (20 vdW interactions and 4 H-bonds) and the lowest (17) in the N51A:C complex (14 vdW interactions and 3 H-bonds). In the two remaining complexes wt hAID:5 mC and N51A:5 mC, the numbers of interactions were nearly identical: 19 (16 vdW interactions and 3 H-bonds) and 20 (18 vdW interactions and 2 H-bonds), respectively (for details, see Fig. 5 and Supplementary Table S2). The comparison of individual interaction networks revealed only two crucial differences in the analyzed patterns of enzyme-substrate interactions. The first, caused by N51-to-A substitution, was the elimination of an H-bond that in the complexes containing wt hAID was formed by N51 and the C2 carbonyl group of the heterocyclic ring. The second difference, caused by exchange of C to 5 mC, was the appearance of vdW interactions between the methyl group and W84 and T27 that partially replaced interactions between T27 and C4-C5-C6 existing in the C containing complexes. Notably, the methyl group interacted with W84, which was also involved in temporal stacking with Y114.

**Figure 6:**
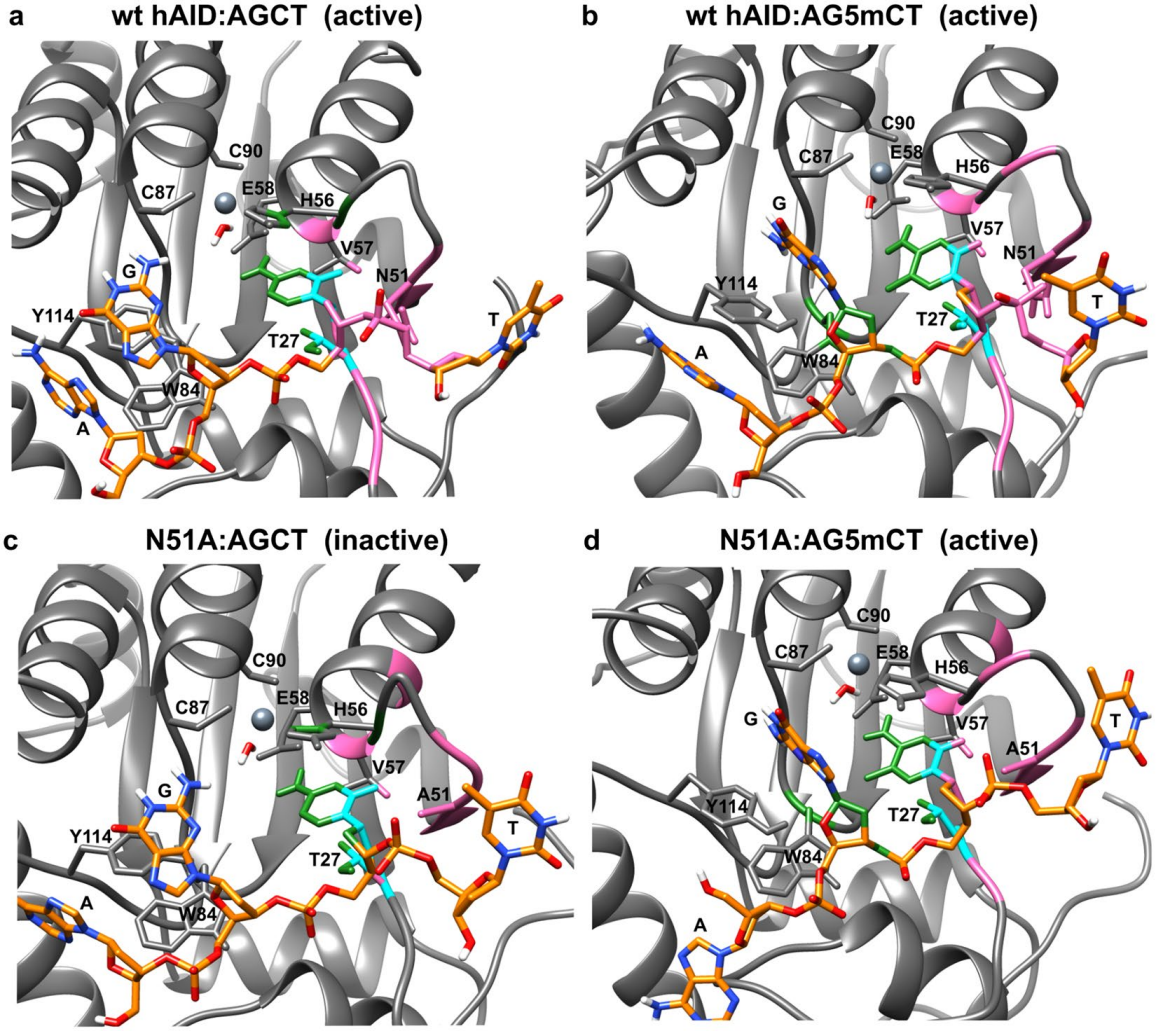
Interactions formed by N51 or A51 residue and methyl group or C5 atom within analyzed complexes: (**a**) wt hAID:AGCT, (**b**) wt hAID:AG5mCT, (**c**) N51A:AGCT, and (**d**) N51A:AG5mCT. The secondary structures of wt hAID and the N51A mutant are shown in gray. The selected amino acid residues that form catalytic pockets of these enzymes are indicated. The dark gray sphere represents zinc ion. The tetranucleotide substrates (AGCT or AG5mCT) docked to the catalytic pockets are shown in orange. The range of interactions of N51 or A51 residue is shown in pink. The range of interactions of the methyl group (of 5 mC) or the C5 atom (of C) is shown in green. The superposition of these two zones is shown in light blue. N51 interacts with an extensive part of the ssDNA sugar-phosphate backbone that joins C and T (within the AGCT/AG5mCT motif). A51 does not form these interactions. Specific to the methyl group are interactions with T27, W84 and an ssDNA sugar-phosphate backbone that joins C and G (within the AGCT/AG5mCT motif). In the N51A:AGCT complex, the ssDNA backbone is not anchored. Stacking interactions between W84, Y114, and G are stable only in complexes with a non-methylated substrate (wt hAID:AGCT, N51A:AGCT).

The second analysis involved complexes formed by the wt hAID/N51A mutant and AGCT/AG5 mCT tetranucleotides. After the manual docking of the latter to the catalytic pockets of wt hAID and the N51A mutant, the complexes were subjected to MD simulations for 50 ns. The simulations confirmed that the N51-to-A substitution significantly decreased the network of enzyme-substrate interactions (Supplementary Table S3). The N51 interacted with the N1-C2 region of the C/5 mC ring, the sugar moiety of C/5 mC, the phosphate group linking C/5 mC to T (within AGCT/AG5 mCT motifs) and the sugar moiety of T. The A51 interactions were limited to the N1-C2 region of the C/5 mC ring and the C1′-O4′ region of the C/5 mC deoxyribose (pink and light blue colors in Fig. 6, Supplementary Table S3). Moreover, N51 differed from A51 in its potential to form H-bonds with the substrate (due to the involvement of HD21/HD22 atoms in hydrogen bonding with deoxycytidine O2, O4′, O3′ atoms and deoxythymidine O2P, O4′, O5′ atoms). In general, the most striking differences between the analyzed patterns of wt hAID:substrate and N51A:substrate recognition concerned extensive N51 interactions with the ssDNA sugar-phosphate backbone (its fragment between C/5 mC and T). The significance of these interactions is emphasized by the fact that AID does not use free cytidine as a substrate^51, 52^.

Subsequently, we compared how the exchange of C into 5 mC affects the network of interactions between the wt hAID/N51A and AGCT/AG5mCT motifs. As shown in Fig. 6 (green and light blue colors) and Supplementary Table S4, the methylation of the substrate resulted in the formation of vdW interactions between the methyl group and T27, W84 and the sugar moiety of G (within the AG5mCT motif). Thus, the methyl group functioned as a bridge between the protein and the sugar-phosphate backbone of ssDNA mediating the anchoring of the latter to the catalytic pocket.

The MD simulations further revealed a stable stacking interaction involving W84, Y114 and the G ring in the wt hAID:AGCT and N51A:AGCT complexes (Supplementary Movies S5 and S7). This stacking could be temporally disrupted in wt hAID:AG5mCT and N51A:AG5mCT complexes or replaced by stacking involving G, Y114 and A (Supplementary Movies S6 and S8). These observations demonstrated that the methyl group significantly influenced the triple stacking system.

## Discussion

AID/APOBEC deaminases have been considered candidate enzymes involved in two out of the three postulated pathways of active genome demethylation^40^. The concept of AID participation in these processes, however, has been challenged by a number of observations. In the case of *in vitro* experiments, several reports have demonstrated the decreased affinity of the AID catalytic center for 5-substituted cytidines compared with that for the non-modified substrate^6, 27, 37^. To better understand structural determinants of hAID activity on different substrates, we tested how selected mutations in hAID affect its ability to deaminate C, 5 mC, and 5 hmC.

As a result, we found that all tested hAID variants were active on 5 mC and had no detectable activity on 5 hmC. Thus, our results confirmed earlier reports demonstrating that 5 hmC is not deaminated by hAID^37, 39^ *in vitro*. In addition, the collected data indicated that the 5-substitution of C with methyl does not prevent—and, in the case of one variant (N51A mutant), increases—the efficacy of hAID-catalyzed deamination. Interestingly, N51A mutant activity on C was below the detection level. This result was further confirmed in the tests involving ssDNAs containing all possible NNCN motifs. In this test we observed robust activity of wt hAID on substrates containing multiple C residues and undetectable activity of the N51A mutant on these substrates.

Notably, we observed that each individual substitution affected hAID’s ability to deaminate C and 5 mC in a different way. Based on the demonstrated decoupling of the hAID deamination activity on C and 5 mC, particularly evident for the N51A mutant, we hypothesized that significant differences in the molecular mechanisms involved in hAID’s recognition of C- and 5 mC-containing substrates must exist. To test this hypothesis we generated wt hAID and N51A models, as well as models of their complexes with substrates which were further refined with extensive MD simulations. Recently, however, an X-ray structure of the highly modified AID variant – AIDv(Δ15) (fusion with MBP protein, 15 substitutions and 3 deletions at the N-terminus, which together reduced the overall charge of AID from +11.9 to +3.4, and a 15 amino acid deletion at the C-terminus) has been published (PDB ID: 5JJ4)^53^. In order to verify the correctness of our models we compared them with the new crystal structure of AIDv(Δ15) using the LGA (Local-Global Alignment) server^54^. All performed tests confirmed high accuracy of our models (see Supplementary Fig. S5). The global root-mean-square deviations of atomic positions (RMSD) calculated for the crystal structure and our models were 1.38 and 1.30 [Å] for wt hAID and N51A variant, respectively. Importantly, structures of the catalytic center and Loop3 (where N51 residue is located) in our models and in the crystal structure were practically identical (see Supplementary Fig. S5b); e.g. RMSDs calculated for Loop3 present in the crystal structure and in our models were 0.218 [Å] and 0.291 [Å] for the wt hAID and N51A variant, respectively. Main differences were observed for Loop1 (the amino acid sequences of this loop were different in our models and in the X-ray structure because of modifications introduced to the crystallized AID variant) and Loop7. These two loops, however, were highly dynamic during the MD simulations, and conformations of both loops observed in the rigid crystal structure are included in the set of conformations predicted in our MD simulation. Thus, we came to the conclusion that our computational models were very accurate, thereby validating our approach, making them suitable to explain the experimental data.

It had previously been proposed that steric hindrance is one of the factors that may decrease the deamination efficiency of the methylated substrate. Our analysis of the models of hAID:substrate complexes showed that both wt hAID and its N51A mutant have enough space in the catalytic pocket to accommodate C and 5 mC. Therefore, steric effects are not the factors that shape the efficacy of C and 5 mC deamination by hAID. Our further *in silico* analysis suggested that at least three elements determine whether C or 5 mC is deaminated. The first element is the network of interactions between the catalytic pocket and the pyrimidine ring that position the latter. However, these interactions are not sufficient for the hAID-catalyzed reaction to occur, which explains why the enzyme cannot deaminate a free nucleoside. The second element is the network of interactions between hAID and the ssDNA sugar-phosphate backbone. Notably, for C- and 5 mC-containing ssDNA, these networks are arranged in different ways. In the case of C-containing substrates, the network includes the interactions between N51 and the sugar-phosphate backbone of C and T in the AGCT motif. These interactions are indispensable for deamination to occur because their abolishment caused by the N51-to-A substitution resulted in the lack of C deamination. In the case of 5 mC-containing substrates, the methyl group functions as a bridge that additionally links hAID (T27 and W84) to the ssDNA sugar-phosphate backbone (the sugar moiety of G in the AGCT motif). Thus, wt hAID is doubly anchored to 5 mC-containing ssDNA. Notably, the interaction network formed by the methyl group is sufficient for the proper positioning of the 5 mC-containing substrate even in the absence of interactions between N51 and the ssDNA sugar-phosphate backbone, which is why the N51A mutant deaminated 5 mC but not C.

The third element is the stacking interaction between W84, Y114 and G adjacent to C in the AGCT motif (W84:Y114:G). Consistent with recently published data, the stacking involving Y114 may contribute to hot-spot recognition by hAID^55^. Notably, our analyses revealed that the methyl group destabilized the triple stacking system. As a result, alternative stacking patterns between A, Y114 and G (A:Y114:G) formed transiently in MD simulations. This finding suggests that hAID’s affinity towards the WRCY motif may be altered if the DNA is methylated. In general, we observed that each of the three above-mentioned elements (that constitute the network of interactions between hAID and substrates) was different for WRCY and WR5mCY. This finding suggests that both substrates are recognized in a distinct way by this enzyme.

**Figure 7:**
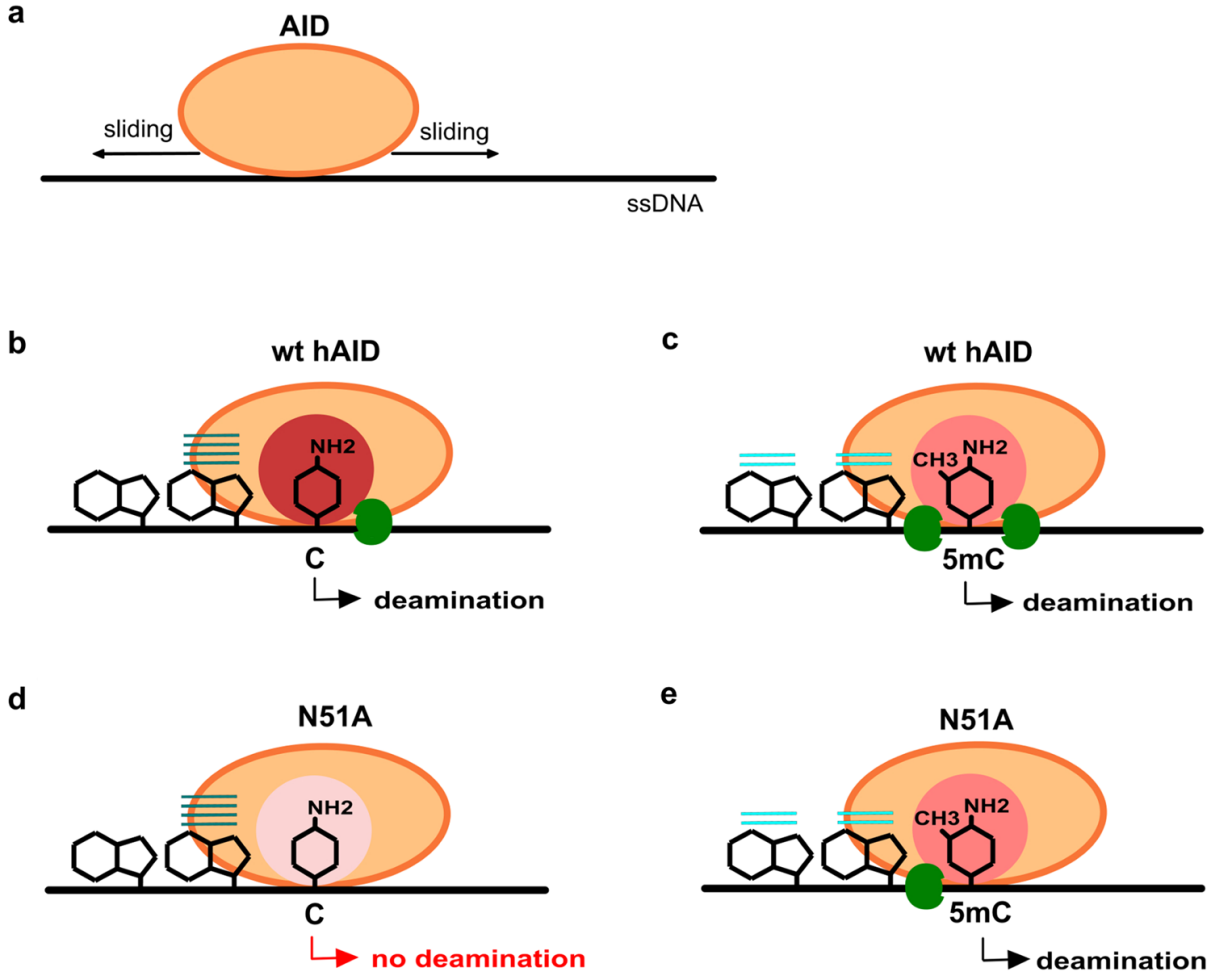
Proposed mechanism of hAID-catalyzed deamination. (**a**) hAID binds to ssDNA and slides bidirectionally along this molecule. (**b**–**e**) Occasionally, the enzyme pauses to deaminate C or 5 mC. There are three elements that determine the enzyme’s propensity to pause: (1) the network of interactions between the catalytic pocket and the pyrimidine ring that position the latter (red circles; the shade of the color corresponds to the strength of the interactions, with darker colors corresponding to stronger interactions); (2) the network of interactions between hAID and the ssDNA sugar-phosphate backbone (one or two green circles); (3) the stacking interactions between the aromatic residues of the protein and nucleobase(s) adjacent to C/5 mC (blue lines; the shade of the color and the number of lines correspond to the stability of the interactions, with darker colors and more lines indicating increased stability. (**b**) The ability of wt hAID to pause and deaminate C is determined by (1) strong stabilization of the pyrimidine ring in the catalytic pocket; (2) N51 residue interactions with the sugar-phosphate ssDNA backbone; and (3) stable stacking interactions (W84:Y114:G). (**c**) The ability of wt hAID to pause and deaminate 5 mC is determined by (1) sufficient stabilization of the pyrimidine ring in the catalytic pocket; (2) double anchoring of the sugar-phosphate ssDNA backbone (by interactions specific to the N51 residue and to the methyl group); and (3) unstable stacking (W84:Y114:G) or alternative stacking (A:Y114:G). (**d**) The N51A mutant failed to deaminate C due to (1) insufficient stabilization of the pyrimidine ring and (2) the lack of an ssDNA backbone anchoring. The stacking (W84:Y114:G) is stable in this case. (**e**) The N51A mutant retains its activity on 5 mC because of (1) sufficient stabilization of the pyrimidine ring and (2) anchoring of the sugar-phosphate ssDNA backbone mediated by the methyl group. In this case, the stacking (W84:Y114:G) is unstable, or alternative stacking (A:Y114:G) is formed.

According to the mechanism proposed by Chelico and coworkers^10^, and more recently by Senavirathne and coworkers^14^, hAID binds and scans (bidirectionally) ssDNA substrates, and it only occasionally pauses to deaminate C. However, this mechanism does not explain what factors cause hAID to pause. Our data suggest that all three aforementioned elements determine the enzyme’s propensity to pause and deaminate C or 5 mC. Based on our findings, we propose the following scenario of hAID-catalyzed deamination (Fig. 7). In accord with the above mentioned model, hAID slides on ssDNA. However, deamination requires the enzyme to be paused (anchored). We propose that the major factor responsible for temporary anchoring hAID to ssDNA is the network of interactions that forms between the protein and ssDNA sugar-phosphate backbone. The lack of this network in the N51A:AGCT complex reduced the enzyme activity on C below the detection level. Moreover, the proposed role of N51 in hAID is consistent with the recently resolved crystal structures of APOBEC3A and APOBEC3Bctd in complexes with substrates^56^. In both complexes the loop3 regions (with N residues corresponding to N51 in hAID) make either direct or water-mediated hydrogen bonds with the phosphate backbone of ssDNA substrate.

An additional factor that may enhance pausing is stacking between aromatic rings of W84, Y114 and rings of bases adjacent to C. In this manner, stacking can also provide specificity in the hAID:ssDNA interaction^55^. Finally, the heterocyclic ring of C/5 mC must be stably placed in the hAID catalytic pocket. Such a situation is observed in the wt hAID:AGCT, wt hAID:AG5mCT and N51A:AG5mCT complexes but not in the N51A:AGCT one. The poorer system of interactions between substrate and catalytic pocket may also contribute to N51A’s inability to deaminate C.

Our models of the hAID:substrate complexes revealed that the methylated substrate is doubly anchored to the wt hAID, whereas the non-methylated substrate is singly anchored. The double anchoring of the enzyme to ssDNA can result in (i) an enhancement of hAID pausing on the substrate, which may increase the deamination rate, and/or (ii) a decrease in the rate of product dissociation, which may slow down the deamination process. The second possibility appears to dominate, which may explain the reported lower efficacy of 5 mC deamination compared with that of C (3-, 10- or 16-fold decrease)^6, 27, 37^. The described decrease in AID activity on the methylated substrate could be regarded as an argument against AID’s involvement in demethylation pathways^37^. However, this decrease does not ultimately exclude the possibility that AID targets methylated substrates *in vivo*. This view is justified if one considers that although AID deaminates C within WRCY hot-spot motifs 4- to 8-fold more efficiently than cold-spot motifs, the deamination of the latter still occurs *in vivo* during SHM^8^. Interestingly, a phosphorylation of the T27 residue (which, according to our results, interacts with the methyl group) has been observed *in vivo* ^57, 58^. Therefore, one can propose that the phosphorylation might affect hAID’s ability to deaminate the methylated substrate. Thus, further *in vivo* studies must be performed to resolve the issue.

The N51A mutant has been described by Honjo’s group as inactive on C *in vitro* ^17^. Unexpectedly, cells harboring this mutant showed half of the wt level of CSR. Because CSR was detected in N51A-cells, the researchers concluded that the DNA deaminase activity does not represent the physiological function of AID^17^. However, they did not examine the possibility that AID targets a modified C. In the context of our data, one can speculate that in N51A-cells, AID deaminated 5 mC in the *Ig locus* and thereby supported CSR. This would mean that AID-dependent 5 mC deamination can play a role not only in the DNA demethylation pathways, but also in other (not yet even identified) biological processes. Nevertheless, there are also some contradictory observations. For instance, Wang *et al*. argued with the finding of Honjo’s group, showing that cells harboring the N51A mutant did not exhibit a detectable antibody diversification^18^. Wang *et al*. confirmed, however, that N51A mutation reduces the *in vitro* deaminase activity of AID on C down to background. Additionally, the authors studied the effect of the N51A substitution on the deamination activity of an up-mutant (which was substantially more active than wt AID), showing that the mutator activity was still detected *in vitro*. Based on this result, the authors suspected that the N51A mutation does not totally destroy AID’s catalytic capabilities. The results presented in our work confirmed this conjecture and suggested that N51A mutation does not affect hAID catalytic center (N51A variant is active on 5 mC) but the N51 residue is involved in the binding of ssDNA substrate. It cannot be also excluded that N51A mutant displays some residual activity on C, however, it was undetectable in our experiments both in UDG-coupled deamination assay and by sequencing method.

Consistent with earlier reports^37, 39^, wt hAID did not show detectable activity on the hydroxymethylated substrate. Moreover, none of the tested mutations affected hAID’s inability to deaminate 5 hmC. The authors of the previous reports proposed that steric hindrance is one of major factors that impedes the binding of 5-substituted C in the hAID catalytic pocket^39^. Based on our data, we propose a novel mechanism that precludes 5 hmC deamination by hAID. This mechanism not only involves exclusion of the hydrated 5 hmC from the catalytic pocket, but also refers directly to the effect of the hydroxyl group on the catalysis.

Although the mechanistic model presented here needs further validation, the knowledge regarding the structural determinants of hAID activity on different substrates may already be used to design and produce AID mutants with new catalytic properties and new potential practical applications. For example, the ability of the N51A mutant to discriminate between C and 5 mC in the long stretches of ssDNA demonstrated in this study may find an application as an alternative to bisulfite treatment in determining the pattern of DNA methylation by sequencing. The production of AID mutants with new catalytic properties appears even more promising taking into account the lately proposed use of an AID ortholog in a CRISPR/Cas9 system^59^. Taken together, our results demonstrate that hAID-mediated 5 mC deamination may be a much more complex phenomenon, than previously expected.

## Methods

### Preparation of recombinant human AID and its mutants

Wt hAID cDNA (GenBank No NM_020661, Genecopoeia) was cloned into the pGEX-4T-1 vector (GE Healthcare) and expressed as a GST fusion protein in *E. coli* strain BL21(DE3)pLysS (Novagen). The expression construct contained sequences encoding GST, a thrombin cleavage site, a HisTag and an enterokinase cleavage site, in that order. Protein production was conducted for 16 h at 18 °C in a *Luria Broth* medium supplemented with 0.06 mM zinc chloride. hAID was purified by using a Glutathione Sepharose 4 FastFlow column (GE Healthcare) according to the manufacturer’s protocol. Fractions containing the recombinant protein were used in biochemical studies of hAID activity. hAID point mutants R50A and N51A were generated by site-directed mutagenesis. The truncated hAID mutant R190X was constructed by PCR amplification. The sequences of all primers are available upon request. All hAID mutants were expressed and purified in the same way as wt hAID. All efforts were made to prevent contamination of the wt enzyme in the preparation of mutants and vice versa (for example, different columns for purification were used).

Some data suggest that the AID recombinant protein previously obtained in a bacterial system might not represent the true activity of AID^60^. To confirm that this is not the case, we performed active-site titration of recombinant wt hAID (see Supplementary Fig. S1) using a method previously described^13, 44^. We performed also a detailed characterization of the recombinant hAID protein obtained by our method, which included MALDI-TOF and western blot analyses, a test of inhibition of hAID’s activity by 1,10-phenanthroline, a test of inhibition of hAID activity by tetrahydrouridine, and a detailed characterization of deamination activity on C-containing and non-C-containing substrates after each step of deamination activity assay described below (all described in Supplementary Methods). Notably, only protein produced at 18 °C in a *Luria Broth* medium supplemented with a source of zinc ions showed enzymatic activity. Most likely zinc ions were necessary for the proper folding of the protein (zinc ions addition after the process, e.g. to the deamination reaction, does not give the same effect and the protein remains inactive), and the low temperature protected bacteria cells against harmful effects of hAID activity. Notably, the deamination activity of the obtained mutants was consistent with characteristics of these mutants previously obtained in other expression systems (see Results and Table 1). In all our biochemical assays reported in this work we used the same four hAID protein preparations, in the same amounts and of similar purity (see Supplementary Fig. S14 in Supplementary Methods).

### hAID activity assays for C, 5 mC and 5 hmC deamination

The uracil-DNA glycosylase (UDG)-coupled deamination assay for measuring AID activity on C has been previously described^43, 45, 46^. Briefly, 0.4 pmol of a ^32^P-5′-labeled 80-nucleotide-long DNA oligomer with a single C residue located in the central position (sequence listed in Supplementary Table S5) was denatured and incubated for 30 min at 37 °C with 4.5 µg of partially purified hAID in 50 mM Tris-HCl buffer, pH 8.0 (a final volume of reaction mixture was 10 µl). In the second stage, two units of *E.coli* uracil-DNA glycosylase (UDG, ThermoScientific) were added to the reaction mixture, and incubation was continued at 37 °C for 30 min. The procedure was followed by incubation for 15 min at 80 °C with a NaOH 0.2 M final concentration to break the DNA strand at the alkali-labile abasic site. The final products of the three-stage reaction were analyzed by electrophoresis in 15% denaturing polyacrylamide gels. In the presence of the deaminase activity, the observation of a radiolabeled 40-nucleotide-long product was expected. For 5 hmC deamination studies, 0.4 pmol of a ^32^P-5′-labeled 80-nucleotide-long DNA oligomer containing 5 hmC instead of C in the central position (Supplementary Table S5) was denatured and incubated for 30 min at 37 °C with 4.5 µg of partially purified hAID in 50 mM Tris-HCl buffer, pH 8.0 (a final volume of reaction mixture was 10 µl). In the second stage of the assay, 5 units of human single-strand-selective monofunctional uracil-DNA glycosylase (hSMUG1, New England Biolabs) were added to the reaction mixture, and incubation was continued at 37 °C for 30 min (modified protocols^37, 61^). The subsequent steps were carried out analogously to the C deamination assay - the reaction mixture was incubated for 15 min at 80 °C with a NaOH 0.2 M final concentration and products were analyzed by electrophoresis in 15% denaturing polyacrylamide gels. Again, in the presence of the deaminase activity, the 40-nucleotide-long product was expected.

For the substrate containing 5 mC (Supplementary Table S5), the previously published assay was applied^38, 47, 62, 63^. Substrate incubation with hAID was followed by annealing with 40 pmol of a fully complementary oligonucleotide to generate a double-stranded DNA with a central G:T mismatch. Annealing was performed in the buffer supplemented with 50 mM KCl. The annealed substrate was incubated for 2 hours at 60 °C with a 4.5 U TDG enzyme (thermostable thymine DNA glycosylase from *Methanobacterium thermoautotrophicum*, Trevigene) in a 1x TDG buffer, followed by alkaline hydrolysis (according to the manufacturer′s recommendation; incubation for 15 min at 80 °C with a NaOH 0.2 M final concentration) and a denaturing 15% PAGE analysis (analogously to the C deamination assay).

As a negative control reaction, untreated with hAID substrate appropriate for each deamination assay was analyzed analogously to the treated samples. As a positive control, an oligonucleotide containing U, 5 hmU or T instead of C, 5 hmC and 5 mC, respectively, (Supplementary Table S5) was used in the corresponding assay. All deaminase assay reactions were carried out in triplicate and analyzed in different gels. The gels were exposed to a phosphor screen, which was scanned using a Fujifilm FLA-5100 Phosphorimager. The band intensities in the individual lanes of each gel were quantified with Multi Gauge software, and an average value for each hAID:substrate combination was calculated.

The degree of dependence between the deaminase activities of the tested hAID variants on C and on 5 mC was calculated as the Pearson product-moment correlation^64^.

Additionally, a number of control reactions were performed for UDG-coupled and TDG-coupled deamination assays (see Supplementary Fig. S8): (i) the control reaction in which, we used the hAID preparation inactivated by 1,10-phenatroline at a concentration of 10 mM (1,10-phenanthroline removes zinc ion from the catalytic center of hAID causing the enzyme to be inactive^6, 43, 45^); (ii) the control reaction in which we used heat-inactivated hAID preparation; (iii) the control reaction in which, instead of the hAID preparation, we used a GST protein preparation. The latter was produced by the expression of the empty vector in the *E. coli* BL21(DE3)pLysS strain (only GST tag was expressed) followed by the analogous purification of the protein extract as in the case of recombinant hAID preparation.

### hAID activity assay for deamination of NNCN motifs

hAID’s ability to deaminate any NNCN motif was tested in four parallel standard reactions, each involving one of four specially designed 104 nt long oligonucleotides; each oligonucleotide contained 16 out of 64 possible combinations of the NNCN motif (sequences are listed in Supplementary Table S5). Briefly, 0.4 pmol of each oligonucleotide was denatured and incubated for 30 min at 37 °C with 4.5 µg of partially purified hAID in 50 mM Tris-HCl buffer, pH 8.0. The final reaction volume was 10 µl. One microliter of the reaction mixture was used as a template in the PCR amplification with KAPA HiFi HotStart Uracil + Polymerase (KAPA Biosystems) according to the manufacturer’s protocol. The procedure was followed by A-tailing, cloning into the pGEM-T Easy vector (Promega) and the sequencing of clones selected by blue-white screening and restriction digestion. Twenty individual randomly selected clones were sequenced per reaction. Subsequently, the obtained sequences were aligned to detect C-to-T transitions. The entire procedure was performed in duplicate.

### Structural models of wild type hAID and its N51A mutant

The structures of wt hAID and N51A mutant were modeled using the SWISS-MODEL homology-modeling server^65, 66^ based on alignments generated by the GeneSilico metaserver^67^ to two structures of the C-terminal catalytic domain of APOBEC3G cytosine deaminase (PDB entries 3V4K and 3E1U) identified as templates. A zinc ion and a water molecule were positioned in the catalytic site of all structures to satisfy the proper tetrahedral structure of the complex. To ensure the stability of the catalytic domain, specific distance restraints of 100 kcal/(mol*Å^2^) were applied to zinc ion, a water molecule, sulfur atoms of Cys87 and Cys90 and a nitrogen atom of His56 such that these moieties were bound to each other.

All generated models were analyzed with Chimera 1.10.1 software^68^.

### Molecular dynamics simulations

All generated structural models were placed in a cubic box with dimensions of 74 × 74 × 74 Å and were subsequently solvated using the TIP3P water model. After solvation, all systems were electrically neutralized by the addition of proper ions, and finally, more ions were added to obtain the physiological concentration of 0.1 M. All following simulations were performed using the NAMD 2.9 software^69^ with the Charmm36 force field^70–72^. All systems were first subjected to potential energy minimization using the steepest descent algorithm and then to solvent equilibration (50 ps) in the canonical (NVT) ensemble at 298 K. Next, 25 ns system equilibration runs were carried out at 298 K using Langevin Dynamics in the isothermal-isobaric (NPT) ensemble with a damping coefficient of 2/ps. Electrostatic interactions were calculated by employing the particle mesh Ewald (PME) method. Finally, based on previously equilibrated systems, production runs of 100 ns were carried out using the same set of parameters as in the equilibration procedure. The structural changes and dynamic behavior of the proteins were analyzed by calculating the RMSD and evolution of distances between selected atoms was monitored.

### Comparison of the AID models with X-ray structure of AID variant (PDB ID: 5JJ4)

We compared the conformations sampled during MD simulation of our AID model with the recently published X-ray structure of AID variant (PDB ID: 5JJ4)^53^. We performed cluster analysis for the obtained trajectory using Chimera software that implements the method described by Kelley *et al*.^73^. Clusters were ranked according to the number of members. Seven clusters that contained the largest number of members were further analyzed. For each of the clusters the average structure was calculated. These average structures were then compared with X-ray structure of AID variant using LGA (Local-Global Alignment) server^54^ (http://as2ts.proteinmodel.org/AS2TS/LGA/lga.html) (see Supplementary Fig. S5). The same analysis was performed for models of both wt hAID and N51A variant.

### Substrate docking

The starting structures of protein models for cytosine bismonophosphate MD runs were generated from frame number 10000 of the wt hAID molecule simulation trajectory. A model of the N51A mutant was generated by truncating the N51 side chain to transform it into an A residue. Therefore, starting structures with essentially identical conformations were generated for all four simulations. In the next step, the C or 5 mC bismonophosphate molecule was manually docked to each of the starting structures. The manual docking procedure was performed in accordance with the proposed mechanism of C deamination^38^. In the C and 5 mC positioning, the most important criteria were the proper location of the C4 atom with respect to the zinc ion, the proximity of the E58 carboxyl group to the amine fragment of cytosine heterocyclic ring (allowing for proton donation to N3) and, in the case of wt hAID model, the proximity of the N51 side chain to the carbonyl group of cytosine. The manual docking of the AGCT and AG5mCT motifs was based on the same criteria. The other criterion included the positioning of the phosphate groups and bases such that the electrostatic repulsion of charges with the same sign would be avoided. After the docking, the complex structure was optimized *in vacuo* using the steepest descent algorithm and the Universal Force Field^74^ implemented in the Avogadro program (http://avogadro.openmolecules.net)^75^. Next, molecular dynamics simulations were performed as described above, except that the duration of the production run was 50 ns.

## Electronic supplementary material

Supplementary Information

Supplementary movie S1

Supplementary movie S2

Supplementary movie S3

Supplementary movie S4

Supplementary movie S5

Supplementary movie S6

Supplementary movie S7

Supplementary movie S8
