# Supplementary material for: Mutations in human AID differentially affect its ability to deaminate cytidine and 5-methylcytidine in ssDNA substrates in vitro
Source: Sci Rep. 2017 Jun 20;7:3873. doi: 10.1038/s41598-017-03936-x (PMC5478644; doi:10.1038/s41598-017-03936-x)
Supplement: Supplementary file 1 — Supplementary Information [file 41598_2017_3936_MOESM1_ESM.pdf]

Supplementary Information

Mutations in human AID differentially affect its ability to deaminate cytidine and 5-methylcytidine in ssDNA substrates *in vitro*

Lucyna Budzko[1]*, Paulina Jackowiak[1]*, Karol Kamel[1], Joanna Sarzynska[1],

Janusz M. Bujnicki[2,3], Marek Figlerowicz[1,4]**

* Joint first authors

** Corresponding author

[1] Institute of Bioorganic Chemistry, Polish Academy of Sciences, Noskowskiego 12/14, 61-704 Poznan, Poland

[2] Laboratory of Bioinformatics and Protein Engineering, International Institute of Molecular and Cell Biology in Warsaw, Trojdena 4, 02-109 Warsaw, Poland

[3] Laboratory of Bioinformatics, Institute of Molecular Biology and Biotechnology, Faculty of Biology, Adam Mickiewicz University, Umultowska 89, 61-614 Poznan, Poland

[4] Institute of Computing Science, Poznan University of Technology, Piotrowo 3A, 60-965 Poznan, Poland

** Corresponding author
Prof. Marek Figlerowicz
Institute of Bioorganic Chemistry
Polish Academy of Sciences
Noskowskiego 12/14
61-704 Poznan, Poland
e-mail: marekf@ibch.poznan.pl

Supplementary Figures……………………………………………………………3

Supplementary Tables…………………………………………………………...17

Supplementary Movies…………………………………………………………..25

Supplementary Methods………………………………………………………... 26

Supplementary References……………………………………………………... 34

Supplementary Figures

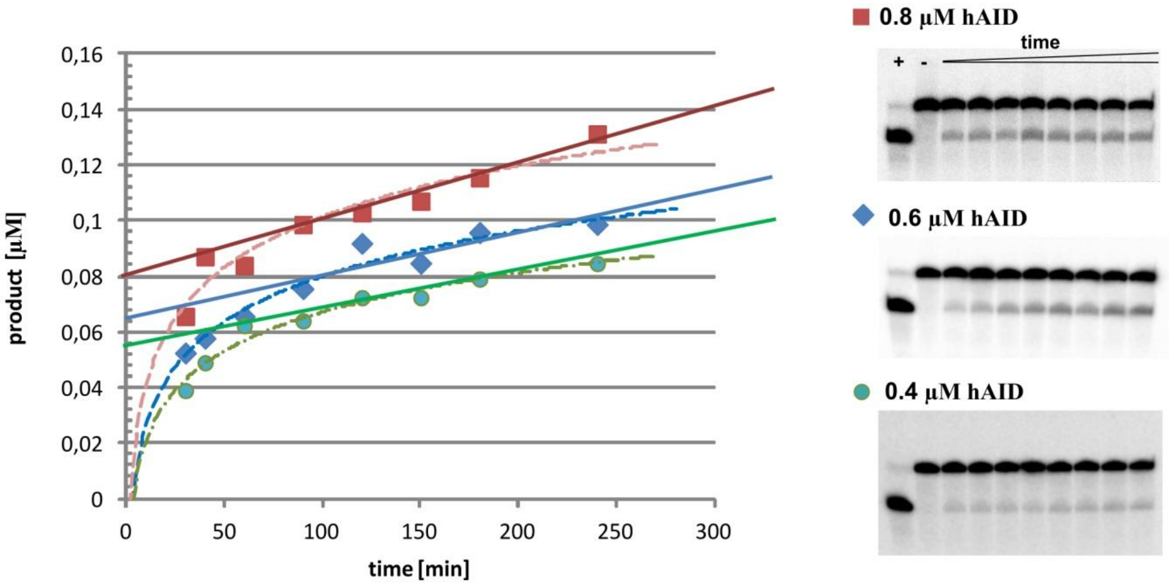

Supplementary Figure S1. Active site titration of wt hAID. Active site titration of wt hAID preparation was performed as previously described[1,2]. Briefly, hAID at three concentrations (0.8; 0.6 and 0.4 µM; the amount of hAID in the protein preparation was estimated based on Coomassie Blue-stained SDS-PAGE gel with Image Lab 5.2 software) was incubated with 5.25 pmol of an unlabeled 80-nucleotide-long DNA oligomer with a single C residue and the same oligomer ^{32}P-5'-labeled used as a tracer. The next steps of the experiment were performed as in UDG-coupled deamination assay (see Methods). Concentrations of the products over time were determined by gel quantitation with Multi Gauge 3.0 software, and plotted as a function of time for different enzyme concentrations. According to the method described by Fersht et al.[3], the concentration of active sites can be determined for steady-state kinetics by taking the linear portion of the chart and extrapolating to determine the y-intercept which is equivalent to $n[E]_0$. Percentage activity of the recombinant protein was then determined by calculating $(active[E]_0/total[E]_0)x100\%$. As a result we found that the percentage of active hAID was 10-14% (average: 11.55 %).

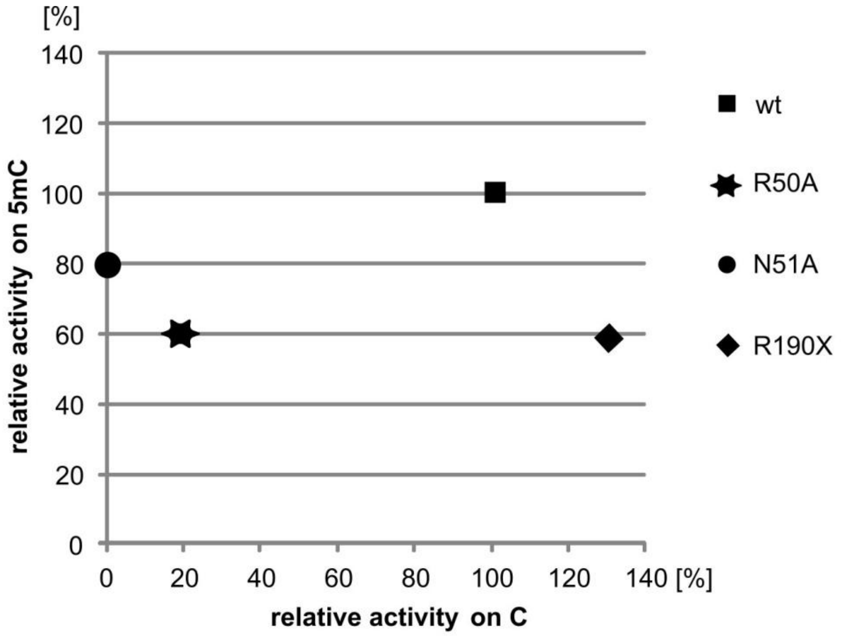

Supplementary Figure S2. Analysis of the correlation between relative activities of the hAID mutants (R50A, N51A, R190X) on C and 5mC. The deaminase activities of the tested hAID mutants were determined as a percentage of the activity of wt hAID (considered as 100%). The calculated linear correlation coefficient was 0.066. The value of a correlation coefficient ranges between -1 and 1. A value of 1 corresponds to a maximum positive correlation, and a value of -1 corresponds to a maximum negative correlation. A value of 0 indicates lack of a correlation.

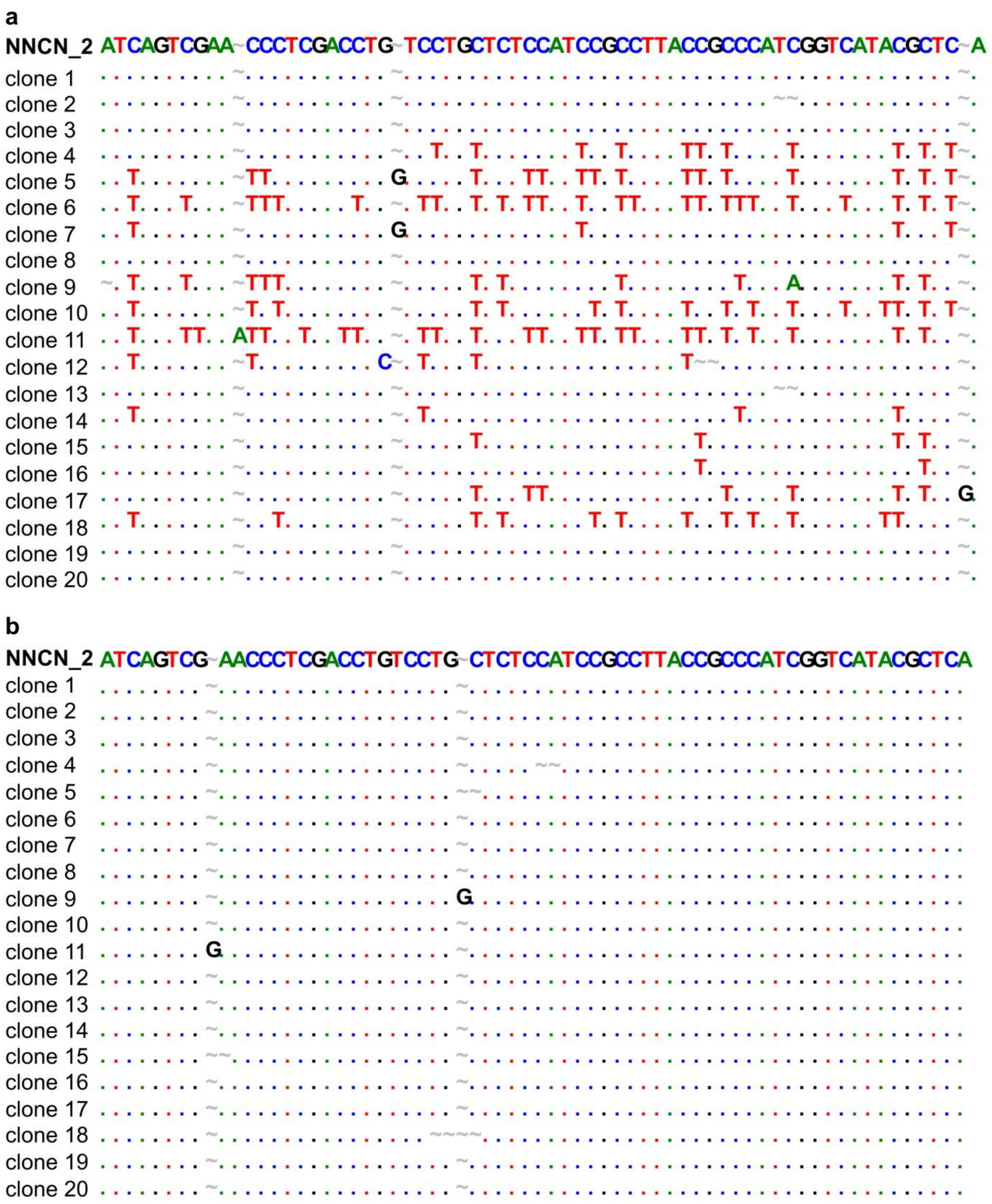
a
NNCN_2 ATCAGTCGAA CCCTCGACCTG TCCTGCTCTCCATCCGCCTTACCGCCCATCGGTCATACGCTC A
clone 1
clone 2
clone 3
clone 4
clone 5
clone 6
clone 7
clone 8
clone 9
clone 10
clone 11
clone 12
clone 13
clone 14
clone 15
clone 16
clone 17
clone 18
clone 19
clone 20
b
NNCN_2 ATCAGTCG AACCCTCGACCTGTCCTG CTCTCCATCCGCCTTACCGCCCATCGGTCATACGCTCA
clone 1
clone 2
clone 3
clone 4
clone 5
clone 6
clone 7
clone 8
clone 9
clone 10
clone 11
clone 12
clone 13
clone 14
clone 15
clone 16
clone 17
clone 18
clone 19
clone 20

c

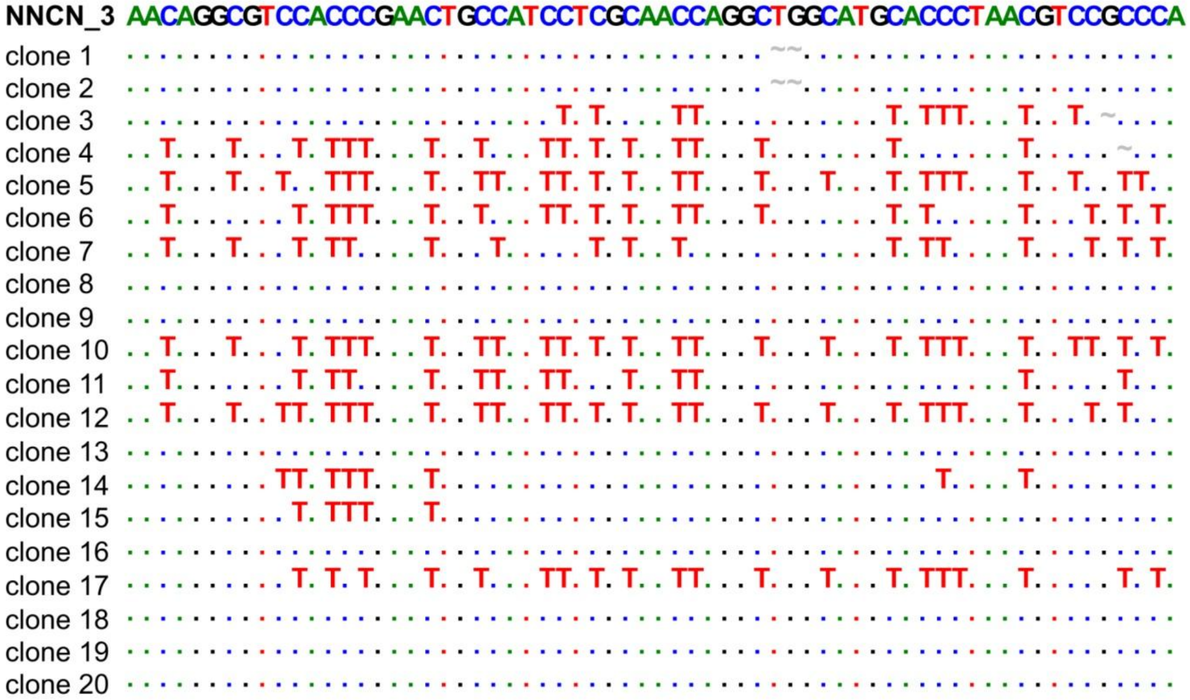

NNCN_3 AACAGGCGTCCACCCGAACTGCCATCCTCGCAACCAGGCTGGCATGCACCCTAACGTCCGCCCA
clone 1
clone 2
clone 3
clone 4
clone 5
clone 6
clone 7
clone 8
clone 9
clone 10
clone 11
clone 12
clone 13
clone 14
clone 15
clone 16
clone 17
clone 18
clone 19
clone 20

d

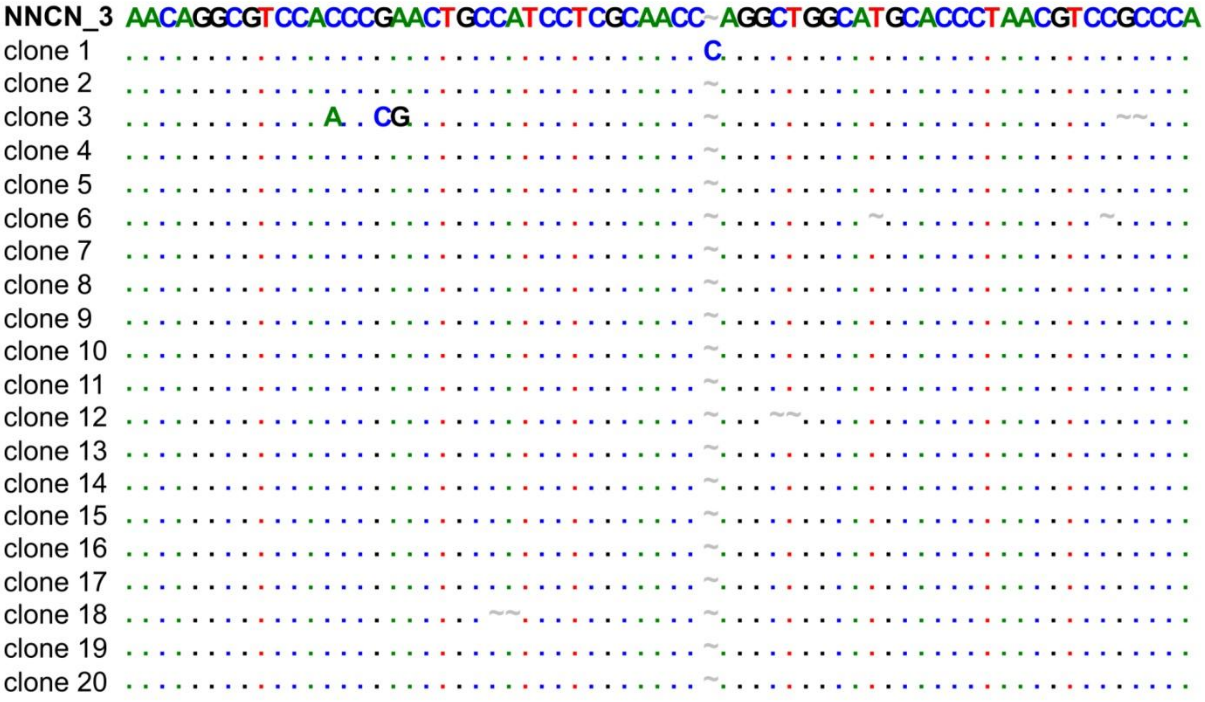

NNCN_3 AACAGGCGTCCACCCGAACTGCCATCCTCGCAACC~AGGCTGGCATGCACCCTAACGTCCGCCCA
clone 1
clone 2
clone 3
clone 4
clone 5
clone 6
clone 7
clone 8
clone 9
clone 10
clone 11
clone 12
clone 13
clone 14
clone 15
clone 16
clone 17
clone 18
clone 19
clone 20

e

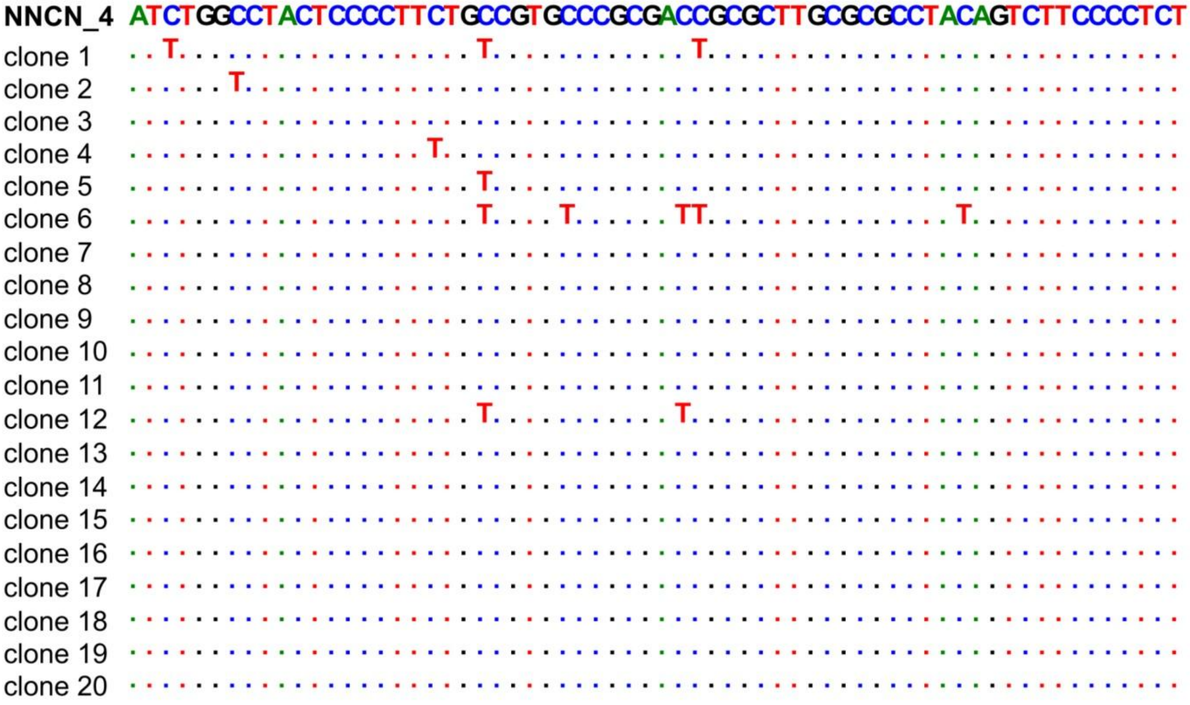
NNCN_4 ATCTGGCCTACTCCCCTTCTGCCGTGCCCGCGACCGCGCTTGCGCGCCTACAGTCTTCCCCTCT
clone 1
clone 2
clone 3
clone 4
clone 5
clone 6
clone 7
clone 8
clone 9
clone 10
clone 11
clone 12
clone 13
clone 14
clone 15
clone 16
clone 17
clone 18
clone 19
clone 20

f

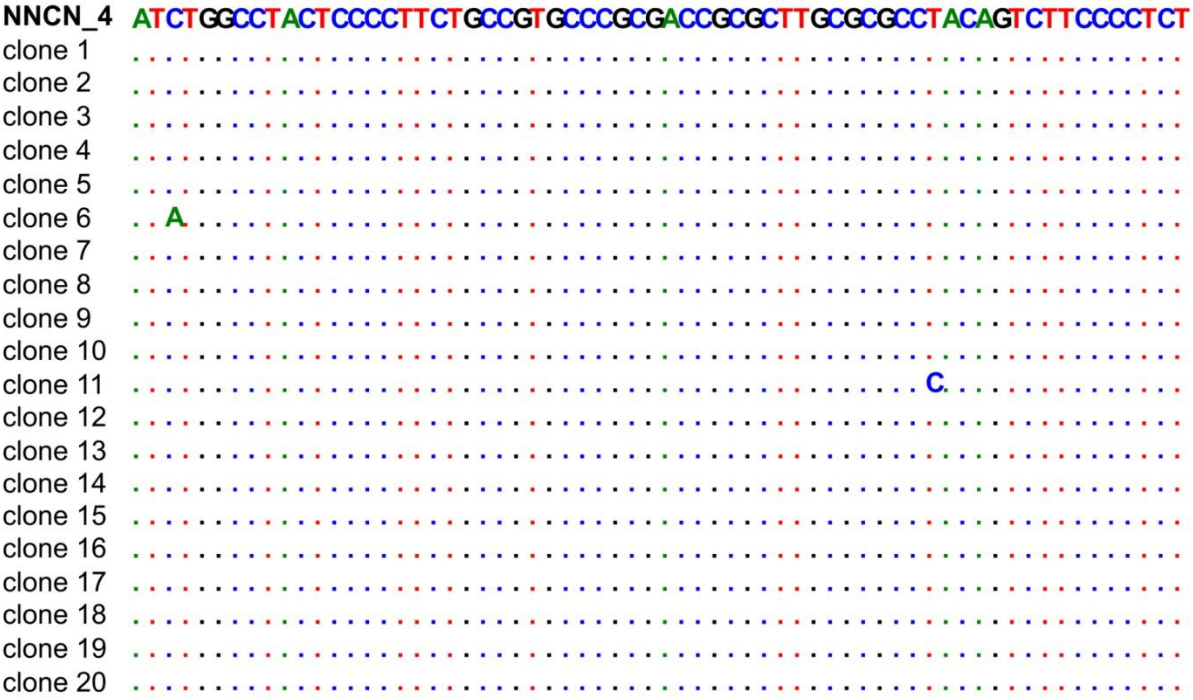
NNCN_4 ATCTGGCCTACTCCCCTTCTGCCGTGCCCGCGACCGCGCTTGCGCGCCTACAGTCTTCCCCTCT
clone 1
clone 2
clone 3
clone 4
clone 5
clone 6
clone 7
clone 8
clone 9
clone 10
clone 11
clone 12
clone 13
clone 14
clone 15
clone 16
clone 17
clone 18
clone 19
clone 20

g

Frequency of deamination of hot spot and cold spot motifs

**motif (5'-3')**	frequency of deamination*
**hot spots**	
AG**C**	0.68
AA**C**	0.42
TA**C**	0.24
TG**C**	0.25
**WRC# average**	**0.40**
**cold spots**	
CC**C**	0.18
CT**C**	0.16
GCC	0.08
GT**C**	0.13
**SYC# average**	**0.14**

***** frequency of deamination defined as the number of times a given motif contains the C to T transition, divided by the number of times the motif occurs (in four tested oligonucleotides and in 20 tested clones)

W = A/T, S = G/C, R = purine, Y = pyrimidine

h **Frequency of deamination of all possible NNCN motifs**

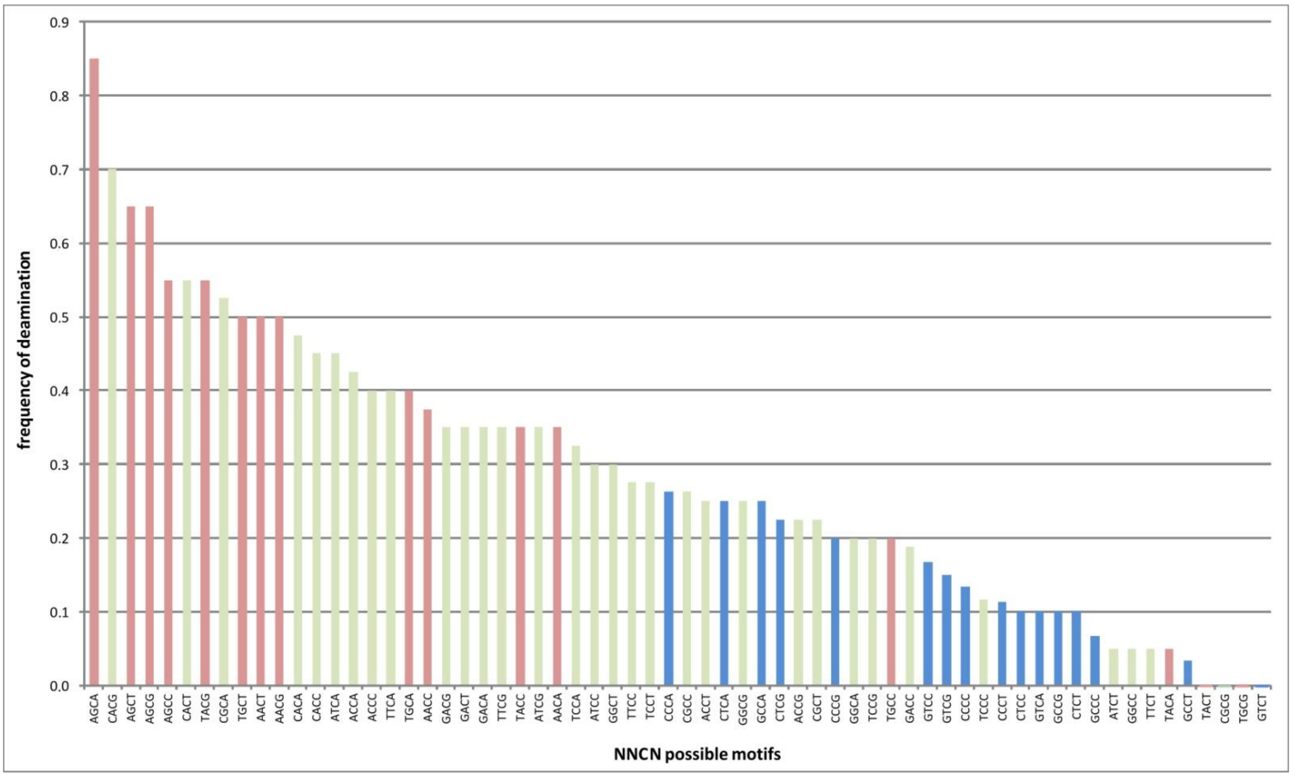

Supplementary Figure S3. Sequence alignments of the 20 obtained clones of NNCN_2 (**a,b**), NNCN_3 (**c,d**) and NNCN_4 (**e,f**) oligonucleotides treated with wt hAID (**a,c,e**) or N51A mutant (**b,d,f**). The first line of each alignment contains the sequence of the NNCN oligonucleotide untreated with hAID. Substitutions are indicated by one-letter symbols corresponding to a particular nucleotide. Positions identical to those in the untreated sequence are marked by dots. Deletions/insertions presumably caused by the used polymerase are marked by "~". Flanking sequences – 20-nt-long PCR primer binding sites are not shown in the figure. (**g**) Frequency of deamination (by wt hAID preparation) of motifs previously published to be hot spots and cold spots for AID[4,5]. (**h**) Frequency of deamination of all possible NNCN motifs. Hot spots are shown in red. Cold spots are shown in blue.

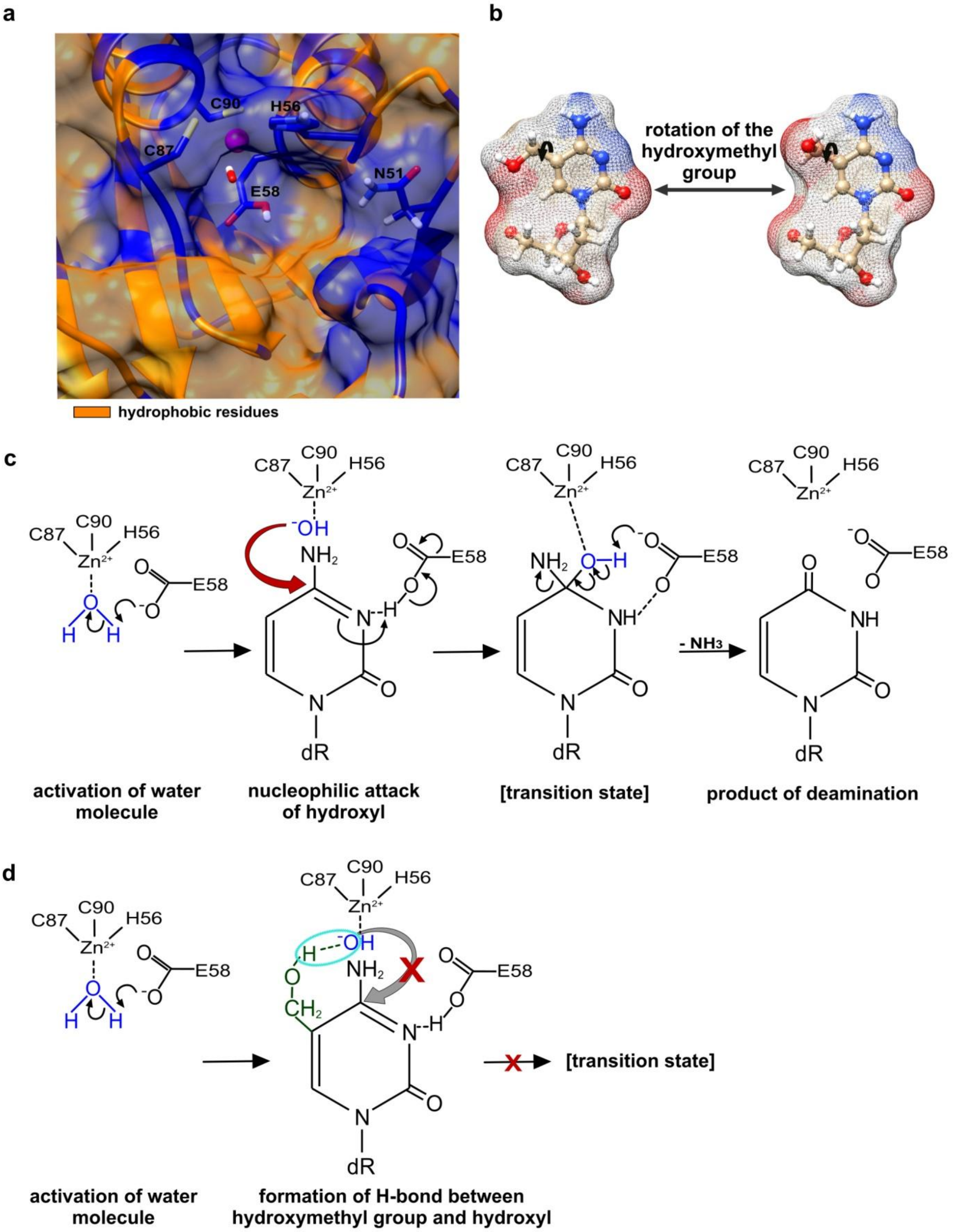

Supplementary Figure S4. Proposed mechanism that prevents deamination of 5hmC by hAID. (**a**) The majority of the hAID catalytic pocket interior is formed by hydrophobic amino acid residues (depicted in orange). (**b**) The possible rotation of the hydroxymethyl group of 5hmC. (**c**) The postulated mechanism of C deamination[6]. This mechanism presumes a direct

nucleophilic attack of the activated water molecule at position 4 of the pyrimidine (red arrow), which leads to the formation of the transition state. (**d**) The possible formation of a H-bond between the hydroxymethyl group of 5hmC and the activated water molecule in the hAID catalytic center (light blue circle). The H-bond disturbs the formation of the transition state and the postulated mechanism of deamination.

a

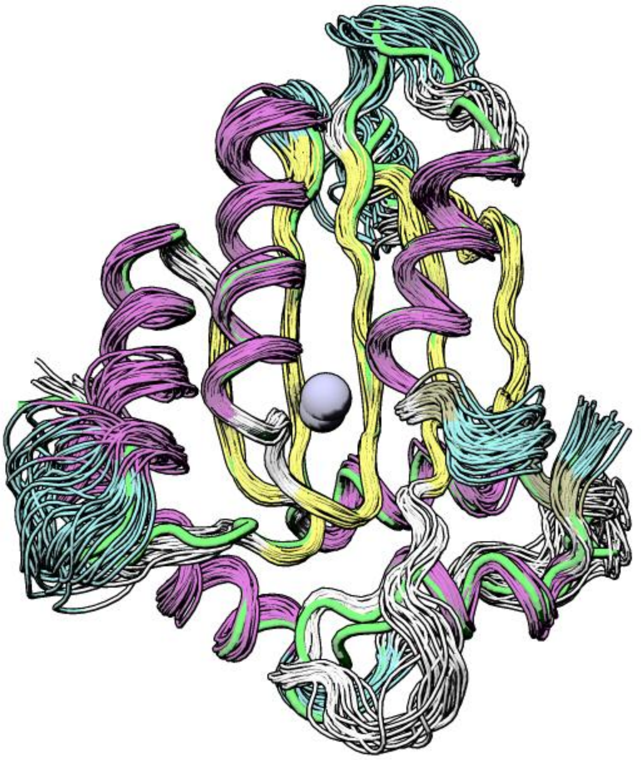

b

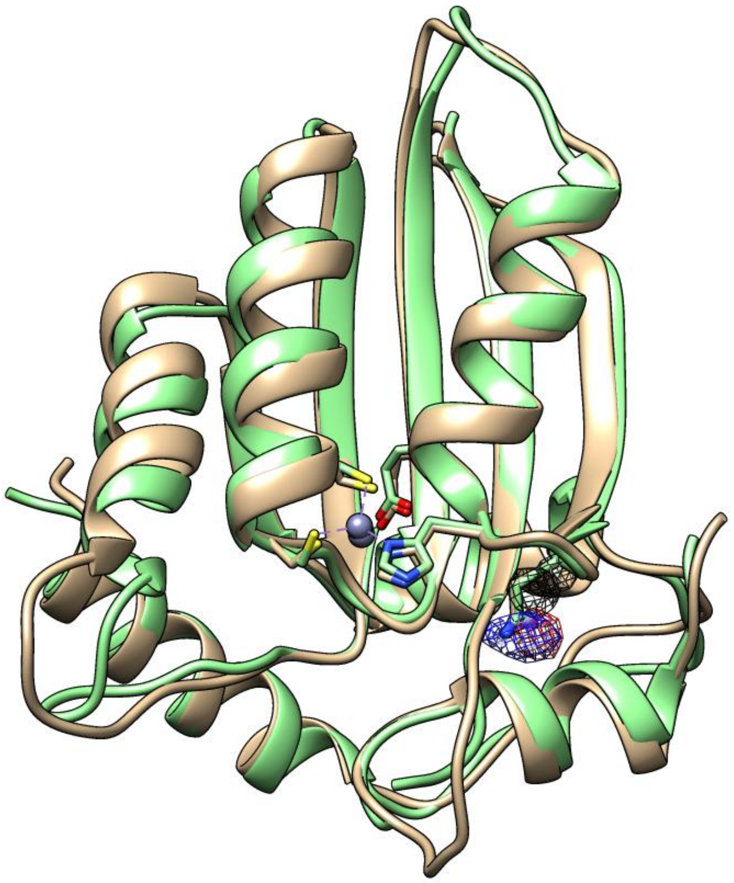

c

selected parameters / compared structures	N1	N2	DIST [Å]	N	RMSD [Å] (for N residues)	GDT_TS	LGA_S	LGA_Q	global RMSD [Å]
wt hAID vs AIDv(Δ15)	172	172	4	166	1.26	89.535	93.056	12.211	1.38
N51A mutant vs AIDv(Δ15)	172	172	4	164	0.99	90.843	93.834	15.037	1.30

N1 - number of residues in our model (only residues 5-19, 23-37, 40-181 that have their counterparts in X-ray structure were analyzed)
N2 - number of residues in X-ray structure
DIST - selected distance cutoff [Å]
N - number of residues superimposed under distance cutoff (DIST)
GDT_TS - Global Distance Test
LGA_S - structure similarity score (0.00 - 100.00)
LGA_Q - quality score

Supplementary Figure S5. Comparison of the generated hAID models with X-ray structure of AID variant - AIDv(Δ15) (PDB ID: 5JJ4). (**a**) Comparison of conformations sampled during MD simulation of the generated wt hAID model (colored by the secondary structure) with the recently published X-ray structure of AID variant (AIDv(Δ15))[7] (shown in green). The gray spheres represent zinc ions. (**b**) Spatial occupancy maps for CA, CB, ND2, and OD1 atoms of N51 residue in the compared structures. The structure of wt hAID model (a representative frame) is shown in beige. The X-ray structure of AIDv(Δ15) is shown in green. The great similarity of compared catalytic centers and Loops3 proves that the generated model is suitable to propose a function of the N51 residue. (**c**) A set of representative parameters calculated by LGA server[8] showing quality of the generated models in comparison with the X-ray structure of AIDv(Δ15).

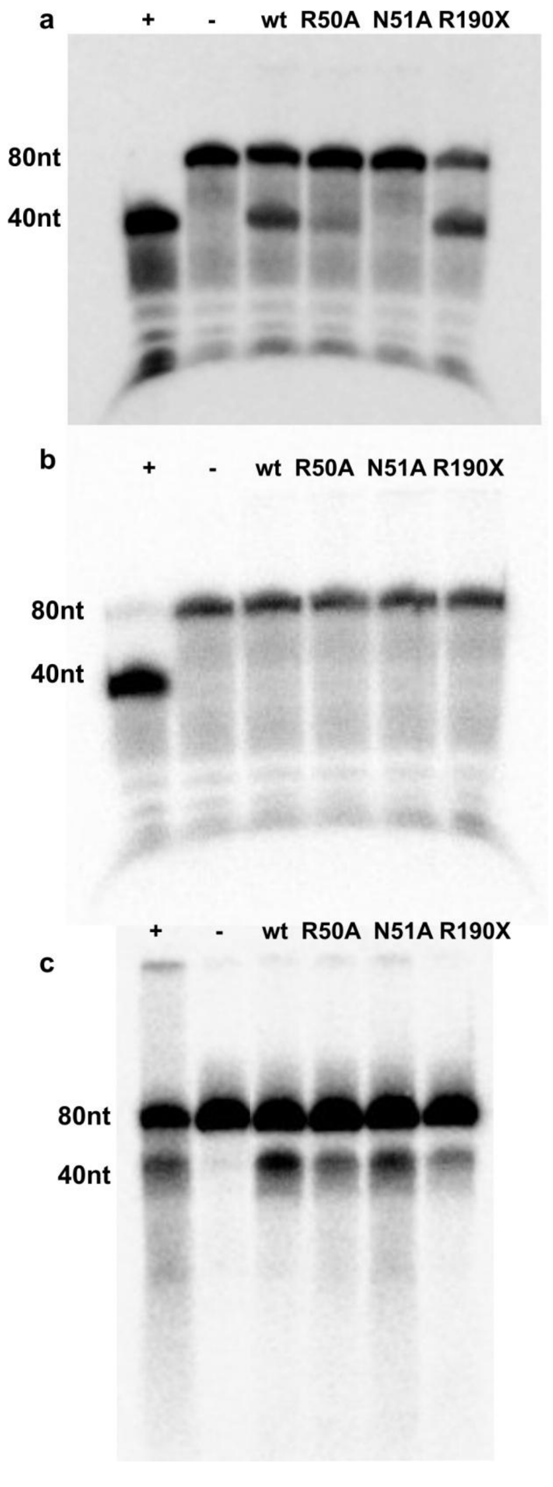

Supplementary Figure S6. Denaturing PAGE analysis of the products of deamination of C (**a**), 5hmC (**b**) and 5mC (**c**). The deaminase activity was tested for hAID variants: wt, R50A, N51A, R190X. Full-length gel images from Figure 1b,e,g (see main text) are presented. In the case of deamination, a 40-nucleotide-long product was expected. Positive and negative control reactions are indicated by “+” or “-”, respectively.

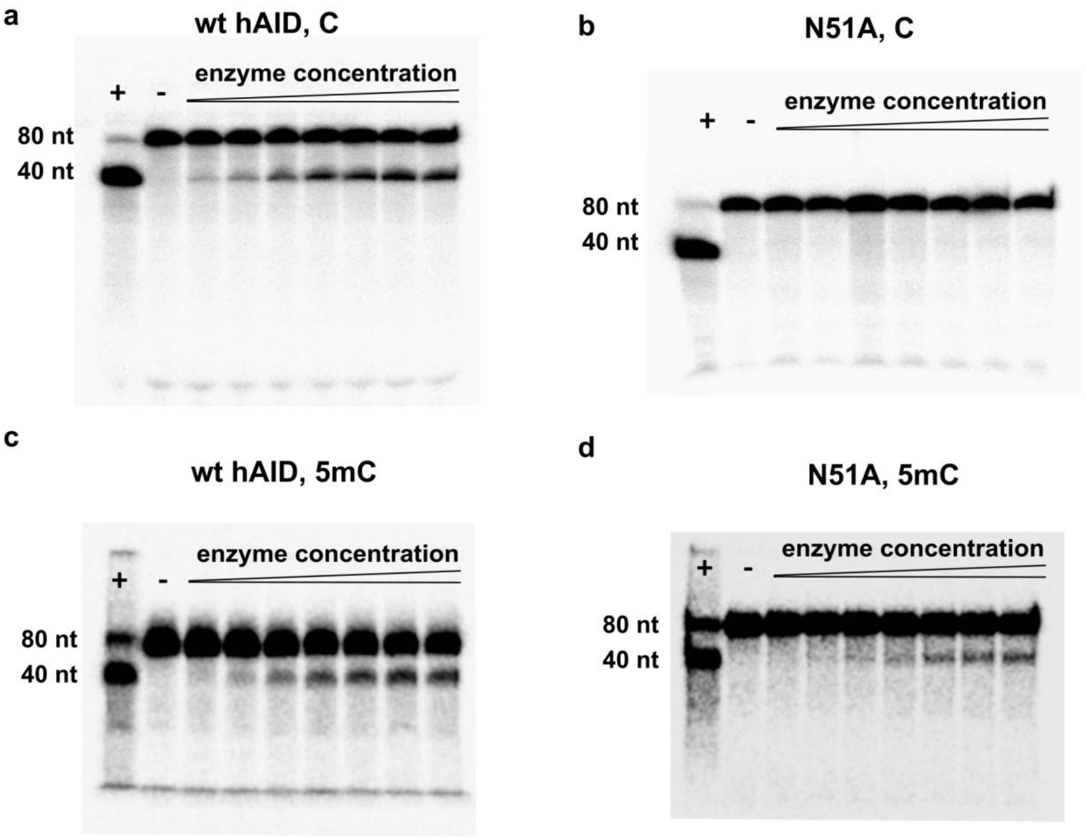

Supplementary Figure S7. Denaturing PAGE analysis of the products of deamination of C (**a,b**) and 5mC (**c,d**) by wt hAID (**a,c**) and N51A mutant (**b,d**). Different concentrations of enzymes were tested. Full-length gel images from Figure 2 (see main text) are presented. In the case of deamination, a 40-nucleotide-long product was expected. Positive and negative control reactions are indicated by "+" or "-", respectively.

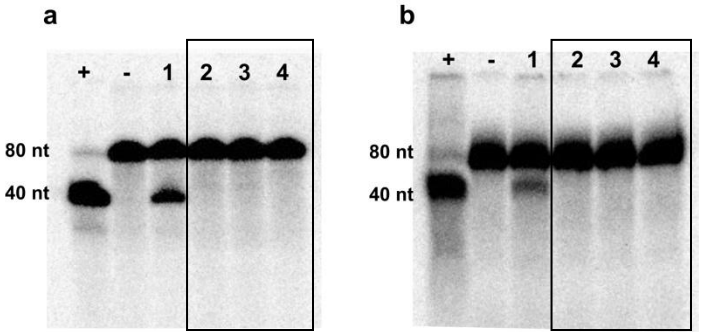

Supplementary Figure S8. Additional control reactions for UDG-coupled and TDG-coupled deamination assays. Denaturing PAGE analysis of the products of deamination of C (a) and 5mC (b) containing substrates. In the case of deamination, a 40-nucleotide-long product was expected. Positive and negative control reactions, untreated with hAID, are indicated by "+" or "-", respectively (for description see Methods). (1) Deamination reactions in which active hAID preparation was used; (2) the control reactions in which we used the hAID preparation inactivated by 1,10-phenatroline at a concentration of 10 mM (1,10-phenanthroline removes zinc ion from the catalytic center of hAID causing the enzyme to be inactive[9-11]); (3) the control reactions in which we used heat-inactivated hAID preparation; (4) the control reactions in which, instead of the hAID preparation, we used a GST protein preparation. The latter was produced by the expression of the empty vector in the *E. coli* BL21(DE3)pLysS strain (only GST tag was expressed) followed by the analogous purification of the protein extract as in the case of recombinant hAID preparation.

Supplementary Tables

Supplementary Table S1. The effect of selected mutations on AID's ability to induce SHM and CSR *in vivo* and to deaminate C *in vitro*.

examples of AID mutants	SHM or CSR efficiency *in vivo* (% in relation to wt AID-cells)		deamination activity on C *in vitro* (% in relation to wt AID)	source
	CSR	SHM		
D45A	30%*	0%	110%	
G47A	20%	0%	5%	
L49A	65%	5%	20%	
**R50A**	**130%**	**25%**	**20%**	[12]
**N51A**	**55%**	**0%**	**0%**	
K52A	130%	15%	80%	
G54A	130%	20%	45%	
**R190X**	**7%**	**75%**	>>100%	[13,14]

*approximate values are given

Supplementary Table S2. Interactions between atoms of C/5mC pyrimidine rings and atoms of amino acid residues that form catalytic pockets of wt hAID/N51A mutant.

residue in the protein model	atoms of the residue	atoms of the substrate in the generated protein/substrate complexes (length of the potential contact [Å][#])			
		**wt hAID:C**	**wt hAID:5mC**	**N51A:C**	**N51A:5mC**
H56	CA*	N3 (3.50)<br>C2 (3.90)<br>O2 (3.54)	O2 (3.52)	-	-
	CB	N3 (3.54)	-	N3 (3.52)	-
	CG	N3 (3.57)<br>C2 (3.78)<br>O2 (3.84)	O2 (3.96)<br>C2 (3.83)	O2 (3.73)<br>C2 (3.72)<br>N3 (3.56)	N4 (3.89)<br>C4 (3.77)<br>N3 (3.47)<br>C2 (3.87)
	CD	N3 (3.80)<br>C2 (3.52)<br>O2 (3.32)	C2 (3.49)<br>O2 (3.40)	C2 (3.50)<br>O2 (3.26)	C4 (3.88)<br>N3 (3.48)<br>C2 (3.50)<br>O2 (3.61)<br>N1 (3.93)
	N	-	O2 (3.94)	-	-
V57	HN	N3 (2.72)<br>O2 (3.84)	O2 (3.86)	N3 (2.68)<br>O2 (3.71)	N3 (1.91)
T27	OG	C6 (3.45)<br>C5 (3.26)<br>C4 (3.62)	C6 (3.71)<br>C5 (3.32)<br>C4 (3.59)<br>C5M (3.49)	C5 (3.29)<br>C4 (3.68)	C5 (3.58)<br>C5M (3.69)
	CB	C5 (3.92)<br>C4 (3.78)	C4 (3.89)	C4 (3.83)	C5 (3.97)
	CG	C4 (3.83)<br>N4 (3.61)	N4 (3.69)	C4 (3.83)<br>N4 (3.60)	C5 (3.46)<br>C5M (3.66)
N51	CB	C2 (3.91)	C2 (3.93)	-	-
	ND	C2 (3.49)<br>N1 (3.85)	C2 (3.40)<br>N1 (3.75)	-	-
	2HD	O2 (2.20)	O2 (2.17)	-	-
A51	CB	-	-	C2 (3.77)<br>O2 (3.33)	C2 (3.40)<br>O2 (3.05)<br>N1 (3.74)
W84	CB	-	C5M (3.95)	-	-
	CD1	-	-	-	C5M (3.62)
Y114	OH	-	-	C5 (3.92)	-

*Designation of atoms is consistent with the nomenclature implemented in Chimera 1.10.1 software.
[#]mean value of distances measured for all individual frames of the MD trajectories

Supplementary Table S3. Atoms within van der Waals distance from the side chain of N51 or A51 residue.

protein:substrate complex	residue (N51/A51), atom of the residue	protein's residue/part of the substrate, atom	distance [Å][#]
wt hAID:AGCT	N51, CB*	V57, CG1	4.0
		T27, CB	4.2
		T27, CA	4.4
		T27, C	4.3
		T27, O	3.4
		T27, N	4.0
		E26, HA	4.4
		L52, N	3.2
		R50, C	3.8
		R50, O	4.3
		**deoxycytidine, O2**	**3.1**
		**deoxycytidine, C2**	**4.0**
		**deoxycytidine, N1**	**4.3**
		**deoxycytidine, C1'**	**3.9**
		**deoxycytidine, O4'**	**3.9**
	N51, CG	T27, HB	4.0
		T27, CA	4.5
		T27, N	3.6
		T27, O	3.7
		E26, C	4.2
		E26, CA	4.0
		E26, HG2	4.2
		R50, C	4.4
		L52, N	3.9
		**deoxycytidine, O2**	**4.4**
		**deoxycytidine, C1'**	**4.2**
		**deoxycytidine, O4'**	**3.8**
		**deoxycytidine, C4'**	**4.5**
	N51, OD1	T27, HB	4.3
		T27, CA	4.2
		T27, C	4.1
		T27, O	3.3
		T27, N	3.1
		E26, C	3.8
		E26, CA	3.5
		E26, CB	3.7
		E26, CG	4.1
		E26, OE2	4.5
		R50, C	4.3
		R50, HB2	3.8
		R50, HE	4.4
		**deoxycytidine, O4'**	**4.5**
	N51, ND2	T27, HN	4.0
		E26, CA	4.3
		E26, HG2	4.3
		R25, O	4.5
		L52, N	4.0
		**deoxycytidine, C1'**	**4.1**
		**deoxycytidine, O4'**	**3.8**
		**deoxycytidine, C4'**	**4.0**
		**deoxycytidine, C3'**	**4.4**

protein:substrate complex	residue (N51/A51), atom of the residue	protein's residue/part of the substrate, atom	distance [Å][#]
wt hAID:AGCT	N51, ND2	**deoxycytidine, O3'**	**3.7**
		**deoxythymidine, O5'**	**4.0**
		**deoxythymidine, C5'**	**3.7**
		**deoxythymidine, O4'**	**4.4**
	N51, HD21	T27, HN	4.1
		E26, CA	3.8
		E26, N	4.5
		E26, CG	4.5
		R25, C	4.4
		**deoxycytidine, O4'**	**4.2**
		**deoxycytidine, C4'**	**4.1**
		**deoxycytidine, O3'**	**4.0**
		**deoxythymidine, O5'**	**3.8**
		**deoxythymidine, C5'**	**3.2**
		**deoxythymidine, C4'**	**3.9**
		**deoxythymidine, O4'**	**3.7**
	N51, HD22	E26, HA	4.1
		L52, O	4.3
		L52, N	3.7
		**deoxycytidine, C1'**	**3.7**
		**deoxycytidine, C2'**	**4.2**
		**deoxycytidine, C3'**	**4.0**
		**deoxycytidine, C4'**	**3.9**
		**deoxycytidine, O4'**	**3.7**
		**deoxycytidine, O3'**	**3.1**
		**deoxythymidine, P**	**4.1**
		**deoxythymidine, O5'**	**3.6**
		**deoxythymidine, C5'**	**3.7**
wt hAID:AG5mCT	N51, CB	V57, CG1	4.0
		T27, CB	4.4
		T27, HG1	4.5
		T27, O	3.9
		T27, HN	3.8
		R50, C	3.7
		R50, O	4.3
		L52, N	3.3
		C55, O	4.3
		**deoxycytidine, O2**	**3.2**
		**deoxycytidine, C2**	**3.9**
		**deoxycytidine, N1**	**4.2**
		**deoxycytidine, C1'**	**4.0**
		**deoxycytidine, O4'**	**3.9**
	N51, CG	T27, CB	4.4
		T27, N	3.8
		T27, HG1	4.2
		T27, O	4.0
		T27, CA	4.5
		E26, HA	3.7
		R50, HB2	4.3
		R50, C	4.2
		L52, N	4.0
		**deoxycytidine, O2**	**4.2**
		**deoxycytidine, C1'**	**4.0**
		**deoxycytidine, O4'**	**3.5**
		**deoxycytidine, C4'**	**4.2**

protein:substrate complex	residue (N51/A51), atom of the residue	protein's residue/part of the substrate, atom	distance [Å][#]
wt hAID:AG5mCT	N51, OD1	T27, CB	3.8
		T27, OG1	4.3
		T27, N	2.8
		T27, C	3.8
		T27, O	3.2
		T27, CA	3.6
		E26, C	3.7
		E26, CA	3.7
		E26, CB	4.3
		R50, HH21	4.5
		R50, CB	4.5
		R50, HA	4.3
		R50, C	4.1
		**deoxycytidine, O4'**	**3.7**
		**deoxycytidine, C4'**	**4.3**
	N51, ND2	T27, HN	3.9
		E26, HA	3.9
		L52, N	4.0
		**deoxycytidine, C1'**	**4.1**
		**deoxycytidine, O4'**	**3.7**
		**deoxycytidine, C4'**	**4.0**
		**deoxycytidine, O3'**	**4.0**
		**deoxythymidine, C5'**	**4.2**
	N51, HD21	T27, HN	3.8
		E26, CA	4.3
		R25, O	4.3
		**deoxycytidine, C1'**	**4.3**
		**deoxycytidine, O4'**	**3.7**
		**deoxycytidine, C4'**	**3.6**
		**deoxycytidine, C3'**	**4.3**
		**deoxycytidine, O3'**	**3.8**
		**deoxythymidine, P**	**4.5**
		**deoxythymidine, O2P**	**4.4**
		**deoxythymidine, O5'**	**4.5**
		**deoxythymidine, C5'**	**3.5**
		**deoxythymidine, C4'**	**4.3**
	N51, HD22	L52, O	4.1
		L52, C	4.2
		L52, CA	4.1
		L52, N	3.4
		**deoxycytidine, O2**	**4.4**
		**deoxycytidine, C1'**	**4.1**
		**deoxycytidine, O4'**	**4.0**
		**deoxycytidine, C4'**	**4.4**
		**deoxycytidine, O3'**	**3.8**
		**deoxythymidine, P**	**4.5**
		**deoxythymidine, O2P**	**4.0**
		**deoxythymidine, C5'**	**4.4**
N51A:AGCT	A51, CB	V57, CG1	3.8
		T27, CB	4.2
		T27, O	3.8
		T27, HN	3.7
		L60, HD11	4.4
		R50, C	3.7
		K52, N	3.3

**protein:substrate complex**	**residue (N51/A51), atom of the residue**	**protein’s residue/part of the substrate, atom**	**distance [Å]#**
N51A:AGCT	A51, CB	N53, HN	3.7
		**deoxycytidine, O2**	**3.0**
		**deoxycytidine, C2**	**3.8**
		**deoxycytidine, N1**	**4.4**
		**deoxycytidine, C1'**	**4.3**
		**deoxycytidine, O4'**	**4.1**
N51A:AG5mCT	A51, CB	V57, CG1	3.6
		T27, CB	4.2
		T27, CA	4.4
		T27, N	3.9
		T27, O	3.7
		L60, HD12	4.0
		R50, C	3.7
		R50, O	4.3
		K52, N	3.4
		C55, O	4.2
		E26, HA	4.2
		E26, HG1	4.2
		**deoxycytidine, O2**	**3.2**
		**deoxycytidine, C2**	**3.9**
		**deoxycytidine, N1**	**4.4**
		**deoxycytidine, C1'**	**4.2**
		**deoxycytidine, O4'**	**3.8**

*Designation of atoms is consistent with the nomenclature implemented in Chimera 1.10.1 software.

#mean value of distances measured for all individual frames of the MD trajectories

Supplementary Table S4. Atoms within van der Waals distance from the methyl group (of 5mC) or C5 atom (of C).

**protein:substrate complex**	**protein's residue/part of the substrate, atom**	**distance from the methyl group [Å]**	**distance from the C5 atom [Å]#**
wt hAID:AGCT	H56, CD2*	—	4.1
	H56, CG		4.3
	T27, CG2		4.1
	T27, OG1		3.7
	T27, CB		4.2
	deoxycytidine, C1'		3.8
	deoxycytidine, C2'		4.3
	deoxycytidine, O4'		4.3
	atoms of the cytosine ring		<4.5
wt hAID:AG5mCT	T27, CG2	4.2	—
	T27, OG1	4.2	
	W84, CB	4.0	
	W84, CG	4.4	
	W84, CD1	4.3	
	S83, HD1	4.1	
	deoxyguanosine, O3'	3.9	
	deoxyguanosine, C2'	4.2	
	deoxyguanosine, C1'	4.3	
	atoms of the cytosine ring	<4.5	
N51A:AGCT	H56, ND1	—	4.4
	H56, CG		4.5
	T27, CG2		4.0
	T27, OG1		3.7
	T27, CB		4.2
	deoxycytidine, C1'		3.7
	deoxycytidine, C2'		4.2
	deoxycytidine, O4'		4.2
	atoms of the cytosine ring		<4.5
N51A:AG5mCT	T27, CG2	4.1	—
	T27, OG1	3.9	
	W84, CB	4.1	
	deoxyguanosine, O3'	4.4	
	deoxyguanosine, C2'	4.1	
	deoxyguanosine, C1'	4.0	
	deoxyguanosine, N3	4.3	
	atoms of the cytosine ring	<4.5	

*Designation of atoms is consistent with the nomenclature implemented in Chimera 1.10.1 software.

#mean value of distances measured for all individual frames of the MD trajectories

Supplementary Table S5. Sequences of the ssDNA substrates used in the hAID activity assays.

name	sequence 5' – 3'
Oligo_C	GGATTGGTTGGTTATTTGTTTAAGGAAGGTGGATTAAAAG**C**TTAATAAGGTGATGGAAGTTATGTTTGGTAGATTGATGG
Oligo_U	GGATTGGTTGGTTATTTGTTTAAGGAAGGTGGATTAAAAG**U**TTAATAAGGTGATGGAAGTTATGTTTGGTAGATTGATGG
Oligo_5mC	GGATTGGTTGGTTATTTGTTTAAGGAAGGTGGATTAAAAG**[5mC]**TTAATAAGGTGATGGAAGTTATGTTTGGTAGATTGATGG
Oligo_T	GGATTGGTTGGTTATTTGTTTAAGGAAGGTGGATTAAAAG**T**TTAATAAGGTGATGGAAGTTATGTTTGGTAGATTGATGG
Oligo_5hmC	GGATTGGTTGGTTATTTGTTTAAGGAAGGTGGATTAAAAG**[5hmC]**TTAATAAGGTGATGGAAGTTATGTTTGGTAGATTGATGG
Oligo_5hmU	GGATTGGTTGGTTATTTGTTTAAGGAAGGTGGATTAAAAG**[5hmU]**TTAATAAGGTGATGGAAGTTATGTTTGGTAGATTGATGG
Oligo_C-complementary	CCATCAATCTACCAAACATAACTTCCATCACCTTATTAAGCTTTTAATCCACCTTCCTTAAACAAATAACCAACCAATCC
NNCN_1	GGATTGGTTGGTTATTTGTTAG**C**AGA**C**GA**CCCC**A**C**GAG**C**TGA**CC**TT**C**A**C**A**CC**AG**CC**GA**C**TTT**CCC**A**C**TAG**C**GGA**C**ATT**C**G**C**A**C**ATATGTTTGGTAGATTGATGG
NNCN_2	GGATTGGTTGGTTATTTGTTAT**C**AGT**C**GAA**CCC**T**C**GA**CC**TGT**CC**TG**C**T**C**T**CC**AT**CC**G**CC**TTA**CC**G**CCC**AT**C**GGT**C**ATA**C**G**C**T**C**ATATGTTTGGTAGATTGATGG
NNCN_3	GGATTGGTTGGTTATTTGTTAA**C**AGG**C**GT**CC**A**CCC**GAA**C**TG**CC**AT**CC**T**C**G**C**AA**CC**AGG**C**TGG**C**ATG**C**A**CCC**TAA**C**GT**CC**G**CCC**ATATGTTTGGTAGATTGATGG
NNCN_4	GGATTGGTTGGTTATTTGTTAT**C**TGG**CC**TA**C**T**CCCC**TT**C**TG**CC**GTG**CCC**G**C**GA**CC**G**C**G**C**TTG**C**G**C**G**CC**TA**C**AGT**C**TT**CCCC**T**C**TTATGTTTGGTAGATTGATGG

20-nt-long PCR primer binding sites within oligonucleotides NNCN_1 – NNCN_4 are underlined.

Supplementary Movies

Supplementary Movies S1 – S4. MD simulations of the generated models of wt hAID (Supplementary Movies S1 and S2) and N51A mutant (Supplementary Movies S3 and S4) with docked deoxycytidine bismonophosphate (Supplementary Movies S1 and S3) or 5-methyldeoxycytidine bismonophosphate (Supplementary Movies S2 and S4). The enzymes are represented by a gray surface with the secondary structure visible. The bismonophosphates docked to the catalytic pockets are shown in orange. The magenta sphere represents the zinc ion. The side chains of selected amino acids residues (C87, C90, H56, E58, P86, Y114, W84, N51/A51) that form the catalytic pockets of the enzymes are shown. The water molecule in the catalytic center is indicated and colored by heteroatoms. The video files were recorded using Chimera 1.10.1 software.

Supplementary Movies S5 – S8. MD simulations of the generated models of wt hAID (Supplementary Movies S5 and S6) and N51A mutant (Supplementary Movies S7 and S8) with docked AGCT (Supplementary Movies S5 and S7) or AG5mCT (Supplementary Movies S6 and S8) motif. Secondary structure of wt hAID or the N51A mutant is shown in blue. The side chains of selected amino acids residues (C87, C90, H56, E58, Y114, W84, V57, N51/A51) that form the catalytic pockets of the enzymes are shown. N51/A51 amino acid residues are shown in white, Y114 is shown in cyan and W84 is shown in magenta. The tetranucleotide substrates (AGCT or AG5mCT) docked to the catalytic pockets are colored as follows: A – green, G – orange, C or 5mC – yellow, and T – pink. The gray sphere represents the zinc ion. The water molecule in the catalytic center is indicated and colored by heteroatoms. The video files were recorded using Chimera 1.10.1 software.

Supplementary Methods

To confirm that the recombinant hAID obtained in a bacterial system (see Methods, main text) represents the true activity of hAID, a detailed characterization of the protein was performed, which included (i) MALDI-TOF analysis; (ii) western blot analysis; (iii) a test of inhibition of hAID activity by 1,10-phenanthroline; (iv) a test of inhibition of hAID activity by tetrahydrouridine; and (v) a detailed characterization of deamination activity on C-containing and non-C-containing substrates after each step of deamination activity assay.

(i) MALDI-TOF

To confirm that the obtained protein is recombinant hAID, MALDI-TOF analysis of the protein extract after purification on a Glutathione Sepharose 4 FastFlow column was performed. A fragment of the polyacrylamide gel, wherein the protein extract was analyzed, corresponding to 50 kDa was excised (Supplementary Figure S9). The protein was eluted from the gel and analyzed in the Laboratory of Mass Spectrometry at the Institute of Bioorganic Chemistry PAS, Poznan, Poland. The analysis confirmed that the excised fragment contained recombinant hAID (peptides of both GST tag and hAID were detected). Moreover, three other gel fragments were analyzed (corresponding to the molecular weight of 60, 38 and 20 kDa; Supplementary Figure S9). The fragments contained proteins present in the analyzed extract in significant amounts. The MALDI-TOF analysis revealed that the protein with a mass of 20 kDa was a product of degradation of the recombinant hAID. The protein with a mass of 60 kDa was identified as a bacterial chaperon protein. The protein with a mass of 38 kDa was not identified. The analysis confirmed that the obtained protein was recombinant hAID and partially excluded the possibility that the protein extract contained a bacterial deaminase or nuclease.

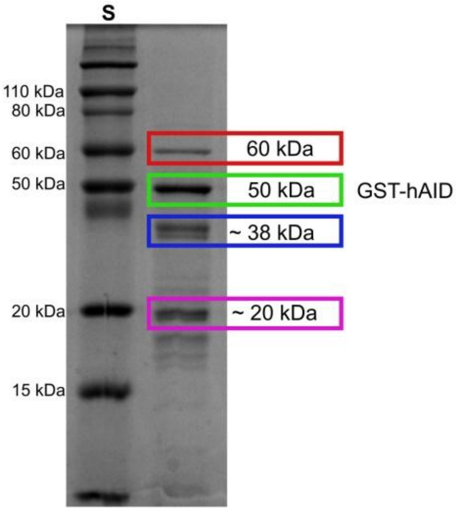

Supplementary Figure S9. Denaturing PAGE analysis of the protein extract obtained after purification on Glutathione Sepharose 4 FastFlow column. Fragments excised from the gel (corresponding to molecular weights of 60, 50, 38 and 20 kDa) are marked by colored rectangles. After elution from the excised gel fragments, proteins were analyzed on a MALDI-TOF mass spectrometer. The full-length GST-hAID fusion protein with the expected mass of 50 kDa is marked by the green rectangle. "S"- protein standard.

(ii) Western blot

Western blot analyses were performed to confirm that the obtained protein extract after purification on the Glutathione Sepharose 4 FastFlow column contained a full-length GST-wt hAID fusion protein with the expected mass of 50 kDa. For the western blot analysis, commercially available primary goat polyclonal anti-AID antibodies (Sigma, No SAB2900301) and secondary rabbit polyclonal anti-goat IgG antibodies, conjugated with alkaline phosphatase (ABCAM ab6742), were used. As presented in Supplementary Figure S10, the western blot analysis confirmed that the obtained fraction contained recombinant hAID.

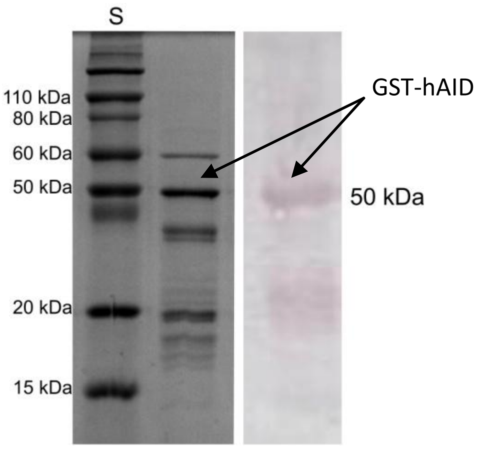

Supplementary Figure S10. Denaturing PAGE and western blot analysis of the protein extract obtained after purification on Glutathione Sepharose 4 FastFlow column. The full-length GST-hAID fusion protein with the expected mass of 50 kDa is marked by arrows. Denaturing PAGE analysis (left) and western blot analysis (right). “S”- protein standard.

(iii) Test of inhibition of hAID activity by 1,10-phenanthroline

1,10-Phenanthroline is a strong complexing factor of the transition metals, which makes it a powerful inhibitor of all zinc-dependent deaminases (including hAID)[15]. The test of inhibition was performed for the protein extract after purification on a Glutathione Sepharose 4 FastFlow column. The reactions were carried out according to the standard hAID activity assay (see Methods, main text), and 1,10-phenanthroline was added to the reaction mixtures at a concentration ranging from 1 mM to 50 mM. As presented in Supplementary Figure S11, the observed deamination activity was inhibited by 1,10-phenanthroline at a concentration of 5 mM or higher. Thus, one can conclude that the observed activity was the activity of the zinc-dependent deaminase.

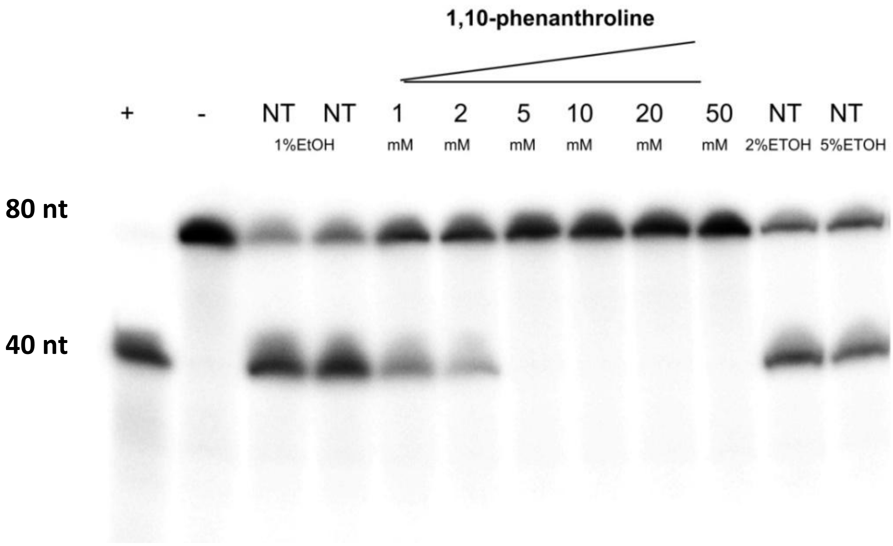

Supplementary Figure S11. Denaturing PAGE analysis of products of the hAID activity assay for C deamination performed in the presence of 1,10-phenanthroline at concentrations ranging from 1 mM to 50 mM. In the case of deamination, a 40-nucleotide-long product was expected. "+" - positive control reaction (the activity assay performed without the protein extract and on ssDNA substrate containing U instead of C in the position of 40th nucleotide); "-" - negative control reaction (the activity assay performed without the protein extract and on ssDNA substrate containing C in the position of 40th nucleotide); "NT 1%ETOH", "NT 2%ETOH", and "NT 5%ETOH" - the activity assay for C deamination performed in the presence of the protein extract, without 1,10-phenanthroline and in the presence of ethanol. 1,10-Phenanthroline is soluble in ethanol. Therefore, control reactions were performed to test the effect of ethanol on the deamination activity. In these reactions, the ethanol was present at a final concentration of 1%, 2% or 5% (5% corresponds to the concentration of ethanol in the reaction containing 1,10-phenanthroline at a concentration of 50 mM). Next lanes - the activity assay for C deamination performed in the presence of the protein extract and 1,10-phenanthroline at concentrations ranging from 1 mM to 50 mM. The deamination activity was completely inhibited by 1,10-phenanthroline at a concentration of 5 mM or higher.

(iv) Test of inhibition of hAID activity by tetrahydrouridine (THU)

THU is an analog of the transition state of the enzymatic reaction catalyzed by deaminases of free cytidine. Therefore, THU inhibits bacterial cytidine deaminase but does not inhibit hAID[6]. The test of inhibition was performed for the protein extract after purification on a Glutathione Sepharose 4 FastFlow column. The reactions were carried out according to the standard hAID activity assay (see Methods, main text) and THU was added to the reaction mixtures at a concentration ranging from 2 μM to 10 mM (it has been shown that bacterial deaminase of free cytidine is inhibited by THU at concentrations ranging from 20 to 50 μM[16]). As presented in Supplementary Figure S12, the observed deamination activity was not influenced by THU. Thus, one can conclude that the observed activity was not the activity of bacterial deaminase.

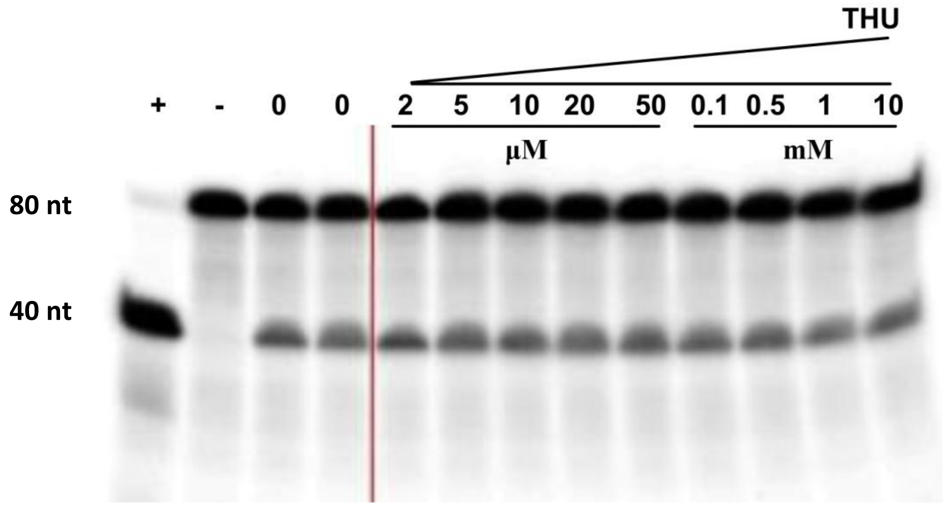

Supplementary Figure S12. Denaturing PAGE analysis of the products of the hAID activity assay for C deamination performed in the presence of THU at concentrations ranging from 2 μM to 10 mM. In the case of deamination, a 40-nucleotide-long product was expected. “+” - positive control reaction (the activity assay performed without the protein extract and on ssDNA substrate containing U instead of C in the position of the 40th nucleotide); “-”- negative control reaction (the activity assay performed without the protein extract and on ssDNA substrate containing C in the position of the 40th nucleotide); “0” - the activity assay for C deamination performed in the presence of the protein extract but without THU; next lanes - the activity assay for C deamination performed in the presence of the protein extract and in the presence of THU at concentrations ranging from 2 μM to 10 mM.

(v) Characteristics of deamination activity on C-containing and non-C-containing substrates after each step of deamination activity assay

The hAID activity assay for C deamination (see Methods, main text) is a three-stage reaction wherein a 40-nucleotide product is generated from an 80-nucleotide ssDNA substrate. It was assumed that the presence of the 40-nucleotide product indicates the presence of deamination activity. To unequivocally confirm that the observed activity is the activity specific to cytidine, the activity of the protein extract on ssDNA not containing cytidine was tested (ssDNA containing T instead of C in the position of the 40th nucleotide was used). The analysis showed no effect of the tested protein extract on the ssDNA molecule not containing cytidine (Supplementary Figure S13d), which proved the reaction specificity. Moreover, products formed after the 1st, 2nd and 3rd stages of the activity assay were analyzed to determine the stage at which the final product appeared. In the case of a nuclease contamination, one could expect the presence of DNA fragments immediately after the 1st stage of the reaction. As shown in Supplementary Figure S13c, only an 80-nucleotide ssDNA substrate molecule was present after the 1st stage of the reaction, which proved that the tested protein extract was not contaminated with nucleases. The appearance of a small amount of 40-nucleotide product after the 2nd stage of the reaction can be explained by the susceptibility of the apyrimidine site (generated in the 2nd stage by UDG enzyme) to damage at high temperature.

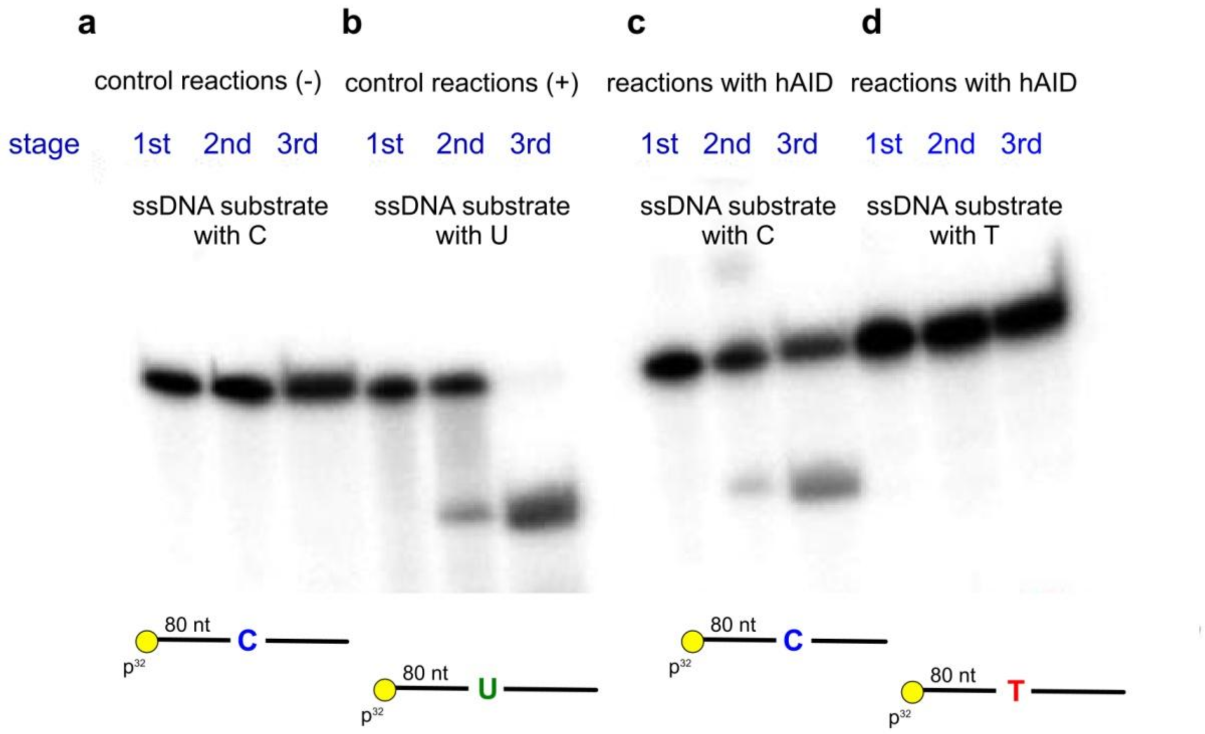

Supplementary Figure S13. Denaturing PAGE analysis of the products of the three-stage hAID activity assay for deamination of C-containing and non-C-containing substrates. Products of the reactions were analyzed after each stage of the assay (1st, 2nd or 3rd stage - shown blue). (**a**) Negative control reactions - the activity assay performed without the protein extract and on an ssDNA substrate containing C in the position of the 40th nucleotide. (**b**) Positive control reactions - the activity assay performed without the protein extract and on an ssDNA substrate containing U instead of C in the position of the 40th nucleotide. (**c**) The activity assay performed in the presence of the protein extract and on an ssDNA substrate containing C in the position of the 40th nucleotide. (**d**) The activity assay performed in the presence of the protein extract and on an ssDNA substrate not containing C (ssDNA substrate containing T instead of C in the position of the 40th nucleotide was used).

Together, the experiments described indicated that the observed deamination activity of the tested protein extract represented the activity of the recombinant hAID.

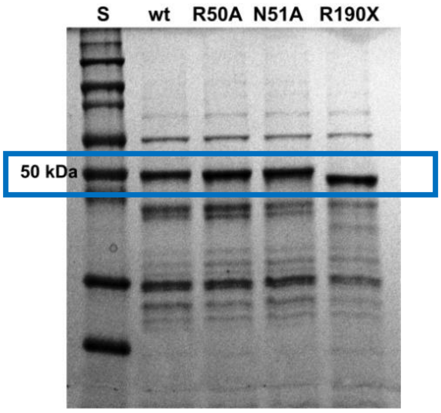

Supplementary Figure S14. Denaturing PAGE analysis of protein preparations of wt hAID and its mutants. The protein preparations were obtained after purification on Glutathione Sepharose 4 FastFlow column. The full-length GST-wt hAID fusion protein (wt) with the expected mass of 50 kDa and its mutants (R50A, N51A, R190X) are marked by blue rectangle. “S”- protein standard.

Supplementary References

1. Coker, H.A. & Petersen-Mahrt, S.K. The nuclear DNA deaminase AID functions distributively whereas cytoplasmic APOBEC3G has a processive mode of action. *DNA Repair (Amst)* **6**, 235-43 (2007).
2. Rangam, G., Schmitz, K.M., Cobb, A.J. & Petersen-Mahrt, S.K. AID enzymatic activity is inversely proportional to the size of cytosine C5 orbital cloud. *PLoS One* **7**, e43279, doi:10.1371/journal.pone.0043279 (2012).
3. Fersht, A.R. et al. Active site titration and aminoacyl adenylate binding stoichiometry of aminoacyl-tRNA synthetases. *Biochemistry* **14**, 1-4 (1975).
4. Bransteitter, R., Pham, P., Calabrese, P. & Goodman, M.F. Biochemical analysis of hypermutational targeting by wild type and mutant activation-induced cytidine deaminase. *J Biol Chem* **279**, 51612-21 (2004).
5. Pham, P., Bransteitter, R., Petruska, J. & Goodman, M.F. Processive AID-catalysed cytosine deamination on single-stranded DNA simulates somatic hypermutation. *Nature* **424**, 103-7 (2003).
6. Samaranayake, M., Bujnicki, J.M., Carpenter, M. & Bhagwat, A.S. Evaluation of molecular models for the affinity maturation of antibodies: roles of cytosine deamination by AID and DNA repair. *Chem Rev* **106**, 700-19 (2006).
7. Pham, P. et al. Structural analysis of the activation-induced deoxycytidine deaminase required in immunoglobulin diversification. *DNA Repair (Amst)* **43**, 48-56 (2016).
8. Zemla, A. LGA: A method for finding 3D similarities in protein structures. *Nucleic Acids Res* **31**, 3370-4 (2003).
9. Sohail, A., Klapacz, J., Samaranayake, M., Ullah, A. & Bhagwat, A.S. Human activation-induced cytidine deaminase causes transcription-dependent, strand-biased C to U deaminations. *Nucleic Acids Res* **31**, 2990-4 (2003).
10. Bransteitter, R., Pham, P., Scharff, M.D. & Goodman, M.F. Activation-induced cytidine deaminase deaminates deoxycytidine on single-stranded DNA but requires the action of RNase. *Proc Natl Acad Sci U S A* **100**, 4102-7 (2003).
11. Wijesinghe, P. & Bhagwat, A.S. Efficient deamination of 5-methylcytosines in DNA by human APOBEC3A, but not by AID or APOBEC3G. *Nucleic Acids Res* **40**, 9206-17 (2012).

12. Shivarov, V., Shinkura, R. & Honjo, T. Dissociation of in vitro DNA deamination activity and physiological functions of AID mutants. *Proc Natl Acad Sci U S A* **105**, 15866-71 (2008).
13. Durandy, A., Peron, S., Taubenheim, N. & Fischer, A. Activation-induced cytidine deaminase: structure-function relationship as based on the study of mutants. *Hum Mutat* **27**, 1185-91 (2006).
14. Barreto, V., Reina-San-Martin, B., Ramiro, A.R., McBride, K.M. & Nussenzweig, M.C. C-terminal deletion of AID uncouples class switch recombination from somatic hypermutation and gene conversion. *Mol Cell* **12**, 501-8 (2003).
15. Barnes, C. & Smith, H.C. Apolipoprotein B mRNA editing in vitro is a zinc-dependent process. *Biochem Biophys Res Commun* **197**, 1410-4 (1993).
16. Cohen, R.M. & Wolfenden, R. Cytidine deaminase from Escherichia coli. Purification, properties and inhibition by the potential transition state analog 3,4,5,6-tetrahydrouridine. *J Biol Chem* **246**, 7561-5 (1971).
